# Carbene metal amide photoemitters: tailoring conformationally flexible amides for full color range emissions including white-emitting OLED†
†Electronic supplementary information (ESI) available: Detailed experimental procedures, single-crystal X-ray diffraction data, photophysical and OLED device characterization, and computational details. CCDC 1912300 for **Au1** (Monoclinic), 1912299 for **Au2**, 1911240 for **Au4**, 1911241 for **Au5**, 1911239 for **Au6**, 1911237 for **Au7**, 1911238 for **Au8** contains the supplementary crystallographic data for this paper. For ESI and crystallographic data in CIF or other electronic format see DOI: 10.1039/c9sc04589a


**DOI:** 10.1039/c9sc04589a

**Published:** 2019-11-13

**Authors:** Alexander S. Romanov, Saul T. E. Jones, Qinying Gu, Patrick J. Conaghan, Bluebell H. Drummond, Jiale Feng, Florian Chotard, Leonardo Buizza, Morgan Foley, Mikko Linnolahti, Dan Credgington, Manfred Bochmann

**Affiliations:** a School of Chemistry , University of East Anglia , Norwich Research Park , Norwich , NR4 7TJ , UK . Email: A.Romanov@uea.ac.uk ; Email: m.bochmann@uea.ac.uk; b Department of Physics , Cavendish Laboratory , Cambridge University , Cambridge CB3 0HF , UK . Email: djnc3@cam.ac.uk; c Department of Chemistry , University of Eastern Finland , Joensuu Campus , FI-80101 Joensuu , Finland . Email: mikko.linnolahti@uef.fi

## Abstract

Conformationally flexible “Carbene–Metal–Amide” (CMA) complexes of copper and gold have been developed based on a combination of sterically hindered cyclic (alkyl)(amino)carbene (CAAC) and 6- and 7-ring heterocyclic amide ligands. These complexes show photoemissions across the visible spectrum with PL quantum yields of up to 89% in solution and 83% in host-guest films. Single crystal X-ray diffraction and photoluminescence (PL) studies combined with DFT calculations indicate the important role of ring structure and conformational flexibility of the amide ligands. Time-resolved PL shows efficient delayed emission with sub-microsecond to microsecond excited state lifetimes at room temperature, with radiative rates exceeding 10^6^ s^–1^. Yellow organic light-emitting diodes (OLEDs) based on a 7-ring gold amide were fabricated by thermal vapor deposition, while the sky-blue to warm-white mechanochromic behavior of the gold phenothiazine-5,5-dioxide complex enabled fabrication of the first CMA-based white light-emitting OLED.

## Abstract 2

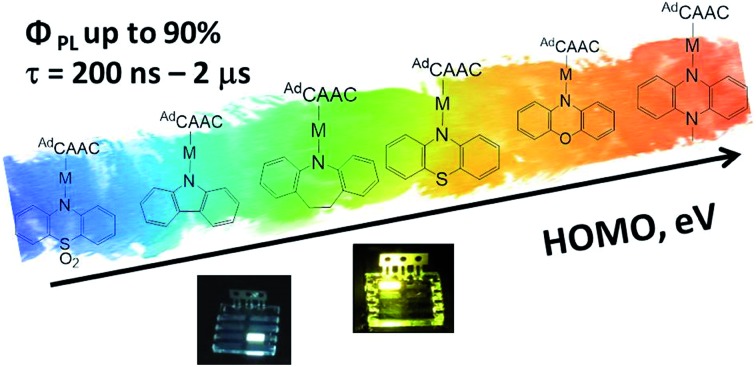
Conformationally flexible “Carbene–Metal–Amide” (CMA) complexes of copper and gold show photoemissions across the visible spectrum, including mechanochromic behavior which led to the first CMA-based white light-emitting OLED.

## Introduction

Two-coordinate coinage metal complexes with linear geometry (L)MX (L = carbene; M = Cu, Ag or Au; X = anionic ligand) have recently emerged as a new class of strongly photoemissive materials.^1^–^4^ Their effectiveness is based on a combination of ligands with complementary donor and acceptor properties: on the one hand, a carbene ligand capable of acting as both a strong electron donor and effective π-acceptor, and, on the other hand, an anionic ligand X that on photochemical or electrical excitation enables charge transfer to the acceptor orbital of the carbene. Cyclic (alkyl)(amino)carbene (CAAC) ligands^5^–^9^ were found to be particularly suitable on the basis of their balance between donor and acceptor properties.^10^ CAAC complexes of copper,^1^ silver^11^ and gold^1^,^7^,^12^ are thermally very stable and resistant to ligand rearrangements. Even simple CAAC copper halide adducts give photoluminescence quantum yields (PLQY) as high as 96%.^1^ Combining CAAC carbene ligands with anions X = arylamide and especially carbazolate proved a particularly effective design strategy for bright phosphors, to give “carbene–metal–amides” (CMAs). The incorporation of such CMA-type copper and gold complexes into the emissive layer of organic light-emitting diodes (OLEDs) enabled the construction of devices with near-100% internal and >25% external quantum efficiency (EQE) by both solution-processing and thermal vapor deposition techniques.^13^,^14^ OLEDs based on mononuclear silver complexes as emitters were obtained similarly.^15^

**Scheme 1:**
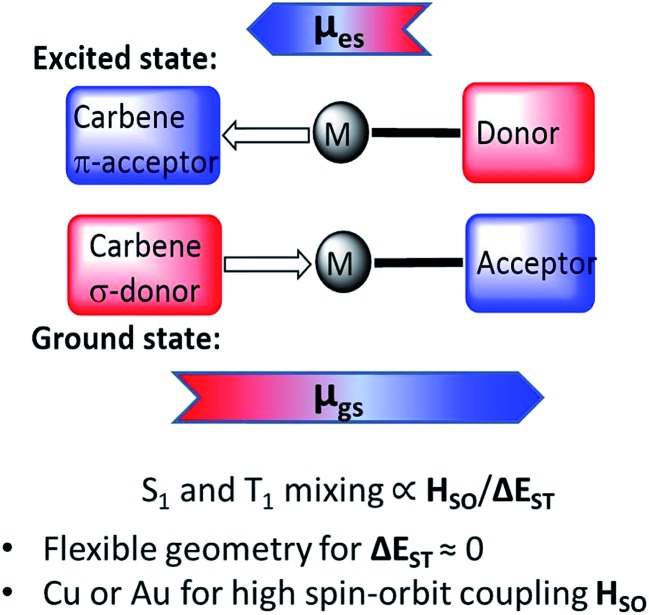
Principle of the CMA structure in the ground and excited states, with arrows indicating direction of molecular ground state (*μ*_gs_) and excited state dipoles (*μ*_es_).

The emission mechanism of CMAs has been the subject of several theoretical and spectroscopic investigations.^16^–^19^ Theoretical calculations revealed that the highest occupied molecular orbital (HOMO) is located mostly on the carbazole, while the lowest unoccupied molecular orbital (LUMO) comprises mainly the C_carbene_ p-orbital.^13^,^15^ Excitation is a ligand-to-ligand charge transfer process (LLCT) from the carbazole to the carbene ligand involving mainly a HOMO → LUMO transition, with only a minor (≤5% to HOMO, 7–15% to LUMO) contribution of the metal orbitals. For gold carbazolate complexes we reported earlier that inter-system crossing to triplets occurs within 4 ps after photoexcitation.^13^ The emission process from CMAs is associated with strong dipole moment changes between ground (*μ*_gs_) and excited states (*μ*_es_) (Scheme 1). This process differs therefore from that operative in most metal-based phosphors where metal-to-ligand charge transfer (MLCT) is dominant.^20^–^28^

The emissions from CMA complexes appear to follow predominantly a thermally activated delayed-fluorescence (TADF) mechanism. However, whereas the classical TADF process shows a characteristic blue-shift on warming,^21^–^24^ CMAs display a temperature-dependent red-shift. Current models suggest that given the rotational flexibility of linear L–M–X complexes, intramolecular twisting of the carbene relative to the amide ligand planes affects both the ground and excited state energy levels. The exchange energy, Δ*E*_ST_, is small and at high twist angles approaches zero.^14^–^18^ This enables efficient mixing of singlet and triplet excited states and results in short (sub-microsecond to microsecond) excited state lifetimes. Radiative rates exceeding 10^6^ s^–1^ and near unity PLQY values have been realized.^13^–^15^ The principle of carbene–metal–amide photoemitters has recently been extended to dendritic systems^29^ and to a range of related carbazolate-based CMA systems.^30^–^32^

Whereas the studies so far have concentrated on the carbazole ligands, where the amide N-atom is locked into a rigid 5-membered ring, we report here a series of CMA complexes carrying amide ligands based on various phenoxazine, phenothiazine, di- and tribenzazepine and sulfone derivatives in which the N-atoms are part of conformationally flexible 6- or 7-membered rings. The electron-donor capacity of these amido ligands varies significantly, so that the luminescence wavelengths can be tuned to cover the visible spectrum from blue to deep red.^33^ We report the use of these emitters in OLEDs, including the first case of a device based on a mechanochromic CMA material, an application enabled by the conformational flexibility of the amide ligand.

## Results and Discussion

### Synthesis and structure

**Scheme 2:**
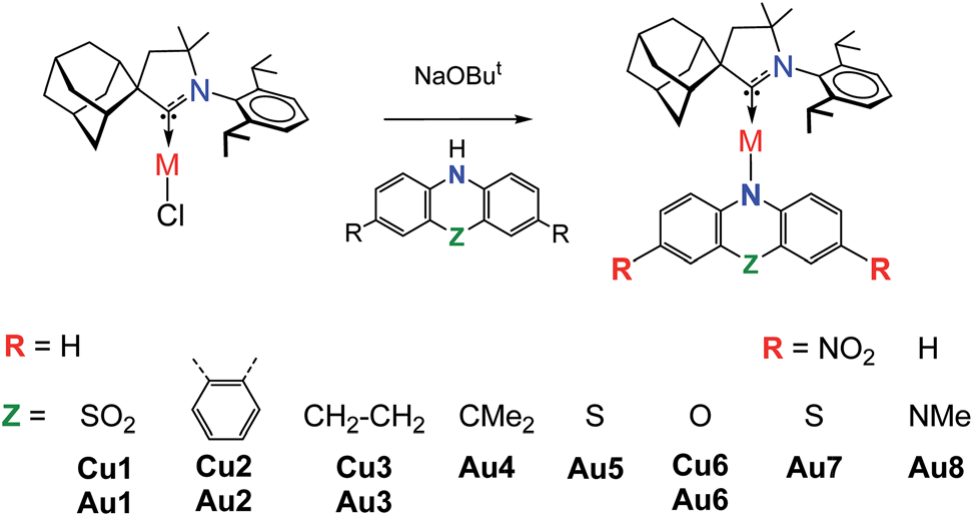
Synthesis and structures of carbene metal amide complexes.

Copper and gold CMA complexes of adamantyl-substituted CAAC ligands (^Ad^L) were prepared in high yields as shown in Scheme 2, following previously established procedures.^2^,^12^,^13^ The color of the complexes in the solid state or in solutions varies significantly and depends on the donor strength of the amide ligand (colorless **Au1**, yellow **Au2–Au4**, orange **Au5**, red **Au6** and deep-purple **Au7**/**Au8**; the copper analogues show very similar colors). The complexes possess good solubility in aromatic and polar non-protic solvents like dichloromethane, THF, and acetone, and moderate solubility in acetonitrile. Gold CMA complexes are stable in air for several months and indefinitely stable under argon. The stability of the copper complexes is reduced to several hours in air as the electron donor strength of the amide ligand increases. According to thermogravimetric analysis (TGA, under nitrogen) the decomposition temperature (*T*_d_) for gold complexes is 15–30 °C higher than for copper compounds (see ESI, Fig. S1†). In the series of gold complexes **Au1–Au8** thermal stability decreases with increasing electron donor properties of the amide ligand, for instance, *T*_d_ is 322 °C for **Au1***vs.* 250 °C for **Au8**.

**Fig. 1:**
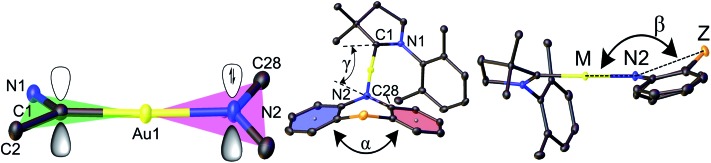
Structural definitions of the dihedral (butterfly) angle *α* between the two best planes of the aromatic 6-rings and deviation of the bridgehead (Z) from the linear CMA unit, angle *β*.

Crystals of the copper and gold complexes suitable for X-ray diffraction were obtained by layering of CH_2_Cl_2_ or toluene solutions with hexane. Key structural parameters for the crystal structures are defined in Fig. 1 and collected in Table 1. The crystal structures are shown in Fig. S2 (see ESI).†

**Table 1:** Selected bond lengths [Å] and angles [°] of copper and gold amide complexes. The values for the monoclinic form of **Au1** are the average for the two independent molecules in the unit cell

	M–C1, (Å)	M–N2, (Å)	C1···N2, (Å)	Angle, (°) C1–M–N2	Torsion angle, (°) N1–C1–N2–C28	N2 deviation from M1···C28···C39, (Å)	Angles, (°) *α*/*β*
**Au1**	1.994(6)	2.055(5)	4.049(6)	176.5(2)	17.4(5)	0.037(6)	154.5(1)/153.7(2)
**Au2**	1.973(3)	2.015(2)	3.988(3)	172.4(1)	23.3(2)	0.074(3)	124.3(1)/138.0(1)
**Au4**	1.984(4)	2.053(3)	4.037(4)	178.73(14)	16.6(5)	0.005(4)	174.8(1)/173.6(1)
**Au5**	1.983(2)	2.043(2)	4.026(2)	177.05(8)	12.9(4)	0.098(2)	144.8(2)/160.3(1)
**Au6**	1.989(6)	2.033(6)	4.021(6)	176.5(3)	21.1(4)	0.070(7)	167.6(2)/160.5(3)
**Au7**	1.992(3)	2.055(3)	4.047(3)	173.6(1)	17.3(3)	0.052(3)	162.1(2)/152.5(1)
**Au8**	1.991(3)	2.032(3)	4.022(6)	177.2(1)	15.6 (3)	0.003(3)	161.7(1)/159.1(1)

All compounds are monomeric, with the molecules arranged into three-dimensional networks by weak C–H···X hydrogen bonds (X = Cl, O, N, and S). There are no close metal–metal contacts. Complexes with six-membered amide ligands (**Au1**, **Au4–Au8**) exhibit C(1)_CAAC_–Au bond lengths that are very similar to the carbazolate complex (^Ad^CAAC)Au(carbazolate) (**CMA1**), while the Au–N(2) bond lengths are longer by 0.01–0.03 Å (Table 1). The carbene-carbon C(1) lies in the N(1)–Au–C(2) plane for all complexes. The amide N-atom N(2) can tend towards a pyramidal geometry (deviation from the M1···C28···C39 plane), as found in phenothiazine-based **Au1**, **Au5**, **Au7**, and in the oxazine **Au6** complexes (Fig. S1†).

Complex **Au2**, which bears a seven-membered amide, shows both C(1)_CAAC_–Au and Au–N(2) bond lengths shorter by 0.01 Å than **CMA1**. Such deviations in bond lengths lead to the C(1)_CAAC_···N(2) distance being longer for six-membered amide complexes (0.01–0.04 Å) and shorter by 0.03 Å for **Au2** compared with **CMA1** (4.117 Å, see Table 1). The shortest C(1)_CAAC_···N(2) distance for complex **Au2** is accompanied by significant deviation from linear geometry around gold atom and shows the smallest C(1)–Au–N(2) angle (172.4(1)°) in the gold series. The distance between donor and acceptor (C1···N2) plays an important role in the charge transfer step and, together with the carbene/amide twist angle, can be used as a molecular design tool to control the HOMO–LUMO overlap.

The six- and seven-membered amide ligands in **Au1** to **Au8** are non-planar (“butterfly” conformation) and conformationally flexible. To evaluate the deviations from planarity we introduce the folding angle *α* as the angle between best planes through the two benzo-rings of the amide ligand (Fig. 1). The angle *β* is defined as the angle between the M–N(2) vector and Z, where Z is a bridgehead or the centroid of the C34–C39 bond of **Au2**, see Fig. 1. Complex **Au4** exhibits the largest angle *α* and displays an almost planar geometry of the amide ligand (Z = CMe_2_). A bent geometry with smaller *α* angles (154–167°) has been found for Z = S, SO_2_, NMe (Table 1), while the smallest values of *α* and *β* are found for **Au2** due to the sterically demanding *o*-phenylene bridge.

Crystals of **Au1** were obtained in two forms, a monoclinic and an orthorhombic phase. Monoclinic crystals were grown by layering a dichloromethane solution with hexane and are obtained as the solvate, **Au1**·0.5CH_2_Cl_2_. They contain two independent **Au1** molecules in the unit cell, which differ by 10° in the *β* angle, indicating the flexibility of the sulfone bridge in six-membered amide *vs.* the rigid five membered carbazolate ligand in **CMA1** (Fig. S3†). Layering a toluene solution of **Au1** with hexane leads to orthorhombic phase which exhibits a more flattened conformation of the amide ligand (*α*/*β* = 159.7(3)/164.6(6)° for the orthorhombic *vs.* 154.5(1)/153.7(2)° for the monoclinic form, Table 1). These structural variations appear to exercise a strong influence on the photo- and electroluminescence properties of **Au1** (*vide infra*). Unfortunately, systematic twining prevents a detailed discussion of bond lengths and angles of the orthorhombic **Au1** complex.

### Electrochemistry

**Fig. 2:**
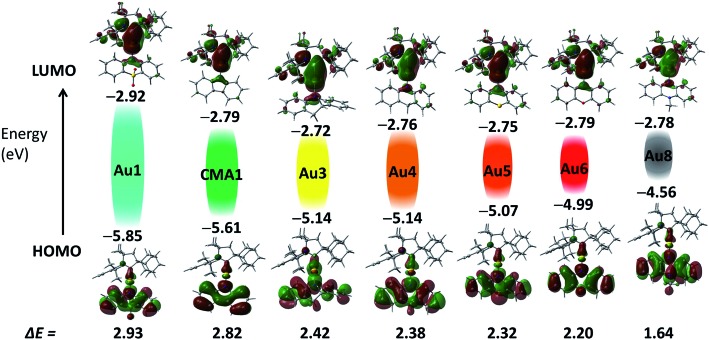
Shapes and energies of HOMOs and LUMOs involved in the vertical excitation (S_0_ → S_1_) of gold complexes **Au1–Au8**, in comparison with the carbazolate complex **CMA1**.^13^ The *E*_HOMO_/*E*_LUMO_ (eV) and band gap values (Δ*E*, eV) were obtained by cyclic voltammetry.

Cyclic voltammetry (CV) was used to analyze the redox behavior of the new copper and gold complexes in MeCN solution, using [^*n*^Bu_4_N]PF_6_ as the supporting electrolyte (Table S1, and ESI, Fig. S4–S10†). All gold complexes show a quasi-reversible, carbene ligand-centered reduction process similar to our previous reports.^2^,^13^,^29^ The peak-to-peak separation Δ*E*_p_ varies within a small range of 77–91 mV for gold complexes; this is higher than the expected value of 59 mV for a theoretical reversible one-electron process. The oxazine complex **Cu6** shows quasi-reversible reduction with a peak-to-peak separation Δ*E*_p_ of 79 mV, whereas other copper complexes **Cu1–Cu3** showed irreversible reduction processes. The lowest reduction potential was found for **M1** (M = Cu and Au) and **Au7** due to the strongly electron-withdrawing nature of the SO_2_ and NO_2_ groups, which results in a stabilization of the LUMO by 0.2 eV for **M1** and 1 eV for **Au7**, respectively (Fig. 2). Unlike **M1** and **Au7**, the reduction potentials of all other complexes are largely insensitive to the nature of the amide ligands and show very similar LUMO energy levels (Table S1,† Fig. 2). Such a marked difference in a first reduction potential for complex **Au7** indicates that it is likely localized on the amide ligand rather than on the carbene (see Fig. S9 in ESI†).

The copper and gold complexes **Cu1–Cu3** and **Au1–Au4** undergo multiple irreversible amide ligand-centered oxidation processes at all scan rates. The phenothiazine-based (**Au5** and **Au7**), oxazine (**Cu6** and **Au6**) and *N*-methylphenazine (**Au8**) show multiple and quasi-reversible oxidation waves with a well-defined back-peak (see ESI, Fig. S5 and S6†). The first quasi-reversible oxidation process shows a peak-to-peak separation Δ*E*_p_ in the range 66–84 mV for copper and gold compounds, whereas larger variations in Δ*E*_p_ values (59–106 mV) were observed for the second or third quasi-reversible processes (Table S1†). The quasi-reversibility of the reduction and oxidation peaks is witnessed by a small shift of 10–20 mV in the peak position *E*_p_ on increasing the scan rate and the peak-to-peak separation Δ*E*_p_ of 77–91 mV (at 100 mV s^–1^), which is close to the ideal value of 59 mV for a one-electron reversible couple. For instance, complex **Au1** shows an increase of the *i*_pa_/*i*_pc_ ratio for the reduction process from 0.74 (at 0.05 V s^–1^) towards unity (0.85 at 2 V s^–1^) which is the ideal value for a reversible couple (Fig. S5, see ESI† for a varied scan rate study for gold and copper complexes showing quasi-reversible redox processes).

On the basis of the electrochemistry data of the free amine ligands^34^ and theoretical calculations all oxidation waves can be assigned to amide-centered processes. The band gap value (Δ*E*, eV see Table S1†) decreases in the series **Au1–Au8** from 2.93 eV for **Au1** to 1.64 V for **Au8** as a result of the decrease in the first oxidation potential and destabilization of the HOMO energy from –5.85 eV for **Au1** to –4.56 eV for **Au8**.^35^ Such trends are in line with the increasing electron-donor strength of the amide ligand. The exception is complex **Au7** which shows a small band gap value of 1.9 eV since in this case both reduction and oxidation processes are centered on the amide ligand, as can be seen by comparison with the free amine ligand 10-*H*-3,7-dinitrophenothiazine (Fig. S9 in ESI†).

### Computational results

The electronic structure of copper and gold complexes was investigated by density-functional theory (DFT) for the ground state and time-dependent DFT (TD-DFT)^36^ calculations for the excited states, using the MN15 functional by Truhlar^37^ in combination with def2-TZVP basis set by Ahlrichs.^38^–^40^ For complexes where X = SO_2_, or S (R = NO_2_) the optimized structures for the S_1_ and T_1_ excited states show twist angles between CAAC carbene and amide ligand planes of only 20°. Excitation of the molecules is accompanied by a flattening of the six- or seven-amide ligands; the *α*/*β* angles are larger by 5–10° compared with the ground state geometries determined by X-ray diffraction.

The LUMO is aligned along the metal–carbene bond, whereas the HOMO is localized on the amide ligand. The contribution of the metal orbitals is ≈3–5% for the HOMO and 7–16% for the LUMO (ESI, Table S3†). The calculated HOMO–LUMO energy gaps are consistent the values obtained by electrochemistry for copper and gold complexes (Fig. 2 and Table S4†). The charge transfer process is predominantly HOMO → LUMO in character (>96%). The copper and gold complexes are characterized by small exchange energies Δ*E*_ST_ of <0.3 eV (Table S5†).

All molecules possess a high ground state dipole moment (*μ*_gs_) in the range of 8–17 D (see ESI, Table S6†) oriented along the metal–C_carbene_ vector. The dipole moments in the excited states (*μ*_es_) are 1–4 D and in most cases changes direction, commensurate with significant charge transfer from the amide to the carbene ligand.

The exception to this is **Au7** where an intraligand HOMO → LUMO+1 charge transfer (ILCT) process for the amide ligand dominates, while the contribution by the HOMO(amide) → LUMO(carbene) transition is low (see ESI, Table S5,†). **Au7** possesses the highest oscillator strength (*f* = 0.2634) and by far the largest exchange energy Δ*E*_ST_ of >0.5 eV, indicative of a potential competing mechanism of ligand-based fluorescence. In agreement with this analysis, **Au7** shows no change in dipole moment since the S_0_ → S_1_ transition is localized on the amide ligand. Evidently, the nitro substituents outcompete the carbene as π-acceptors and stabilize local excited states.

### Photophysical properties

**Fig. 3:**
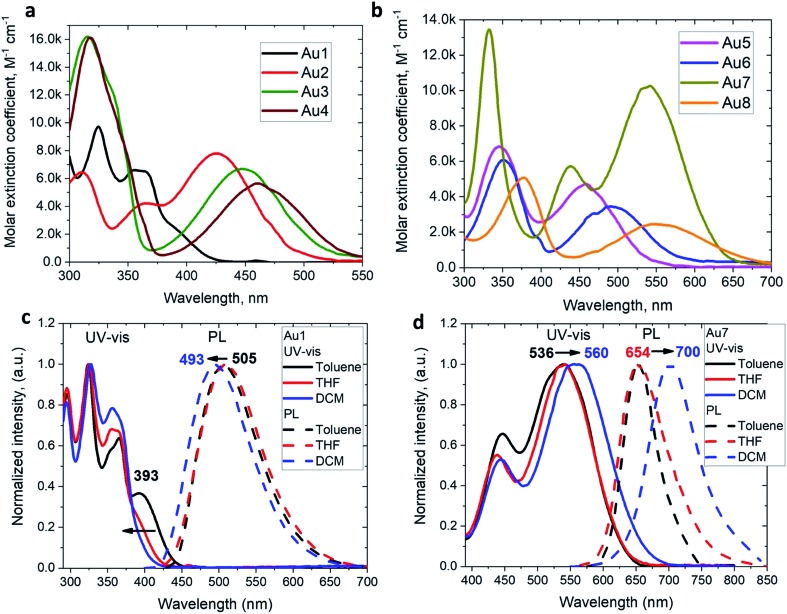
UV/vis spectra of gold **Au1–Au4** (a) and **Au5–Au8** (b) complexes in THF solution (298 K). Normalized UV-vis and emission spectra in toluene, THF and CH_2_Cl_2_ for gold complex **Au1** (c) and **Au7** (d) showing negative and positive solvatochromism, respectively.

The UV/vis absorption spectra for copper and gold complexes in THF solution (Fig. 3 and S11†) show strong π–π* transitions at *ca.* 270 nm ascribed to an intra-ligand (IL) transition of the CAAC carbene. The broad low energy absorption band for copper and gold complexes from *ca.* 380 to 560 nm is assigned to ligand-to-ligand charge transfer L(M)LCT {π(carbazole)–π*(CAAC)}, with a minor contribution of the metal orbitals according to the DFT calculations. The absorption onset of the CT band and peak position shows a red-shift of over 9600 cm^–1^ (*ca*. 175 nm) for the gold series of complexes; this trend follows the increase in electron-donor character of the amide ligands and is largely consistent with the decrease in the band gap values (Δ*E*, Table S1†) identified by cyclic voltammetry (*vide supra*). The molar extinction coefficients of the copper complexes are 2–3 times lower than those of their gold analogues, in line with the theoretically calculated trend in oscillator strength (see ESI Table S5†). The molar extinction coefficients (*ε*) of the L(M)LCT for gold complexes decrease from *ε* = 7800 for **Au2** to 2500 M^–1^ cm^–1^ for **Au8** in THF solution. **Au7** is the exception and shows the highest value of *ε* = 10 260 M^–1^ cm^–1^ due to the dominant contribution of the amide IL π–π* transition.

**Fig. 4:**
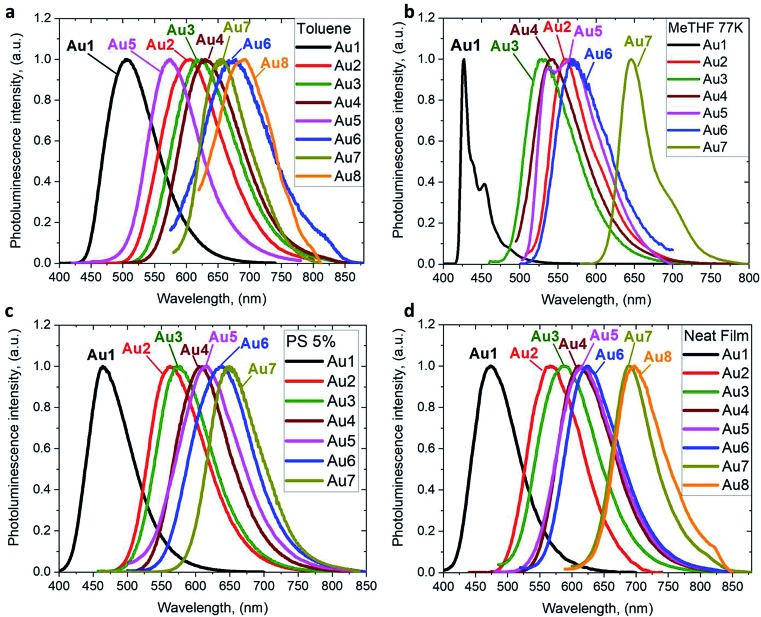
PL spectra of gold CMA complexes **Au1–Au8** in toluene solution at 298 K (a), in MeTHF at 77 K (b), as a 5 wt% dopant in polystyrene (c), and as neat films under N_2_ (d).

The photoluminescence (PL) properties of copper and gold complexes were investigated in toluene solution at 298 K, in frozen 2-methyltetrahydrofuran (MeTHF) at 77 K, as a 5 weight-% dopant in a polystyrene (PS) matrix, and as neat films under N_2_ (Fig. 4; see Fig. S11 and S13† for copper complexes). The PL data are summarized in Table 2. The complexes exhibit broad intramolecular charge transfer emission profiles with FWHM (full-width half-maximum) values of 65–120 nm. The emission colors range from sky-blue to deep-red/near-IR. In a polystyrene matrix, where molecular flexibility is restricted, emissions are slightly blue-shifted relative to the PL in toluene solution, and in the series of gold complexes **Au1–Au7** the PL quantum yields (PLQYs) decrease from 83 to 8% as the wavelength increases. The luminescence of all complexes is reversibly quenched by O_2_. The emission profiles red-shift following the band gap values (Δ*E*, Table S1†), *e.g.* for the gold complexes in the sequence from **Au1** to **Au8**. The basicity of the amine can be correlated with the emission properties of the CMA materials. For instance, the flexible amine ligand 10*H*-phenothiazine 5,5-dioxide (p*K*_a_ = 15.7),^41^ which incorporates a strongly electron-withdrawing linker (Z = SO_2_), reduces the basicity compared with carbazole (p*K*_a_ = 19.9), resulting in sky-blue luminescence for **Au1***vs.* green for **CMA1**. On the other hand, more basic amines with Z = *o*-phenylene (p*K*_a_ = 26.1), ethylene (p*K*_a_ = 25.5), S (p*K*_a_ = 22.7) or O (p*K*_a_ = 21.6)^41^ produce either yellow or orange/red emitters **Au2–Au6** and **Au8**. However, since there is a complicated simultaneous interplay between the structural and electronic factors, the emission colours cannot be predicted solely on the amine basicity. For instance, the amines used for complexes **Au2–Au4** have similar p*K*_a_ values but the emission profile red-shifts by *ca.* 10–20 nm from yellow (**Au2**) to yellow-orange (**Au3**) to orange (**Au4**). This can be rationalized by difference in amide geometry, where the rigid linker Z = *o*-phenylene (**Au2**) leads to the most acute butterfly angles (*α*/*β*), whereas **Au4** shows an almost flat amide ligand (*α*/*β ca.* 180°). The same trend is clear when linkers Z are heteroatoms with comparable rigidity: yellow-orange **Au5** (Z = S) show a butterfly angle *α* which is 20° more acute than in orange-red **Au6** (Z = O). If Z = NMe this trend can be extended into the near-IR range, as in complex **Au8**.

**Table 2:** Photophysical properties of copper and gold complexes as neat films, in toluene solution and in polystyrene (PS) matrix at 5 weight-%

	*λ*_em_ (nm)	*τ* ^*e*^(μs)	*Φ* ^*b*^ (%, N_2_)	*k*_r_ ^*c*^ (10^5^ s^–1^)	*k*_nr_ ^*d*^ (10^5^ s^–1^)	FWHM (nm)	^1^CT/^3^LE^*f*^ (eV)
**Toluene**							
**Cu1**	489	1.13	49.5	4.3	4.4	87	2.85/2.92
**Au1**	505	0.74	89.6	12.1	1.4	94	2.78/2.95
**Cu2**	589	0.58	19.5	3.3	13.8	109	2.44/–
**Au2**	603	0.15	20	13.3	53.3	114	2.38/–
**Cu3**	607	0.43	3.0	0.07	23.1	111	2.37/–
**Au3**	620	0.19	1.7	0.9	51.7	114	2.31/–
**Au4**	629	0.14	2	1.4	70.0	112	2.25/–
**Au5**	574, 665	0.27	0.4	0.14	36.8	96	2.45/–
**Cu6**	661	0.11	—	—	—	121	2.16/–
**Au6**	672	0.25	1	0.4	39.6	126	2.20/–
**Au7**	654	0.025; 0.6	8	—	—	91	2.08/–
**Au8**	689	0.04	<0.1	—	—	109	2.11/–

**Neat film** ^*a*^							
**Cu1**	469	3.3	6.5	0.2	2.8	86	
**Au1**	473	0.71	24.9	3.5	10.5	82	
**Cu2**	524	1.37	26.2	1.9	5.3	101	
**Au2**	566	0.21	11.7	5.5	42.0	98	
**Cu3**	484	1.6	0.1	0.001	6.2	120	
**Au3**	587	0.8	6.4	0.8	11.7	107	
**Au4**	610	0.27	8.1	3.0	34.0	99	
**Au5**	616	2.75	8.6	0.3	3.3	106	
**Cu6**	611	—	—	—	—	99	
**Au6**	624	0.37	—	—	—	98	
**Au7**	690	—	—	—	—	74	
**Au8**	696	—	—	—	—	90	

**5% PS matrix** ^*a*^							
**Cu1**	458	8.52	25.6	0.3	0.8	76	
**Au1**	464	0.87	65.5	7.5	3.9	75	
**Cu2**	528	3.60	36.8	1.0	1.7	114	
**Au2**	563	0.61	39.7	6.5	9.8	95	
**Cu3**	490	4.1	1.7	0.04	2.3	100	
**Au3**	574	1.5	50	3.3	3.3	93	
**Au4**	607	0.15	32.7	21.8	44.8	93	
**Au5**	615	2.82	29.6	1.0	2.5	104	
**Cu6**	614	—	—	—	—	95	
**Au6**	637	0.77	17.2	2.2	10.7	105	
**Au7**	649	2.2	8.5	—	—	90	
**Au8**	—	—	—	—	—	—	

^*a*^Films (neat and in PS host) were prepared by drop-casting from chlorobenzene solutions (10 mg mL^–1^) onto a hot quartz substrate and annealed for 5 min.

^*b*^Quantum yields determined using an integrating sphere.

^*c*^radiative rate constant *k*_r_ = *Φ*/*τ*.

^*d*^Nonradiative constant *k*_nr_ = (1 – *Φ*)/*τ*.

^*e*^In case of two-component lifetime *τ* an average was used: *τ*_av_ = (*B*_1_/(*B*_1_ + *B*_2_))*τ*_1_ + (*B*_2_/(*B*_1_ + *B*_2_))*τ*_2_, where *B*_1_ and *B*_2_ are the relative amplitudes for *τ*_1_ and *τ*_2_, respectively.

^*f*^ ^1^CT and ^3^LE energy levels based on the onset values of the emission spectra blue edge in MeTHF glasses at 77 K and in toluene solutions at 298 K.

The 3,7-dinitrophenothiazine compound **Au7** is an exception in the series: the LUMO contains a significant contribution of the amide ligand, while the LUMO+1 is entirely located on the amide ligand (see ESI, Tables S2 and S4†). As a result excitation of **Au7** leads to intra-ligand HOMO → LUMO+1 transition without flipping of the transition dipole moment value (Table S6†). This is supported by positive solvatochromic behavior in the UV-vis and photoluminescence spectra in solvents of different polarities (see Fig. 3). Au7 differs therefore markedly from the other gold complexes (see Fig. 3 for **Au1** and Fig S15† for **Au2–Au4**) which show a negative solvatochromism due to the flipping of the transition dipole moments between ground and excited states (Table S6†).

The emission spectra were measured in frozen 2-MeTHF solution to identify the energy of the local excited triplet ^3^LE state. All complexes exhibit a significant blue-shift upon cooling to 77 K (Fig. 4b). Complexes **Cu1** and **Au1** show well-structured emission profiles originating from the ^3^LE state of the amide ligand. Complexes **M2–M6** (M = Cu, Au) show only blue-shifted broad CT emissions (see Fig. 4b) upon cooling, which preclude identification of the ^3^LE energy and the Δ*E*(^1^CT–^3^LE) energy gap values. A similar behavior has recently been reported for copper carbazolate complexes with mono- and diamidocarbene ligands.^31^ Unlike **M1–M6**, the emission profile for **Au7** is not shifted upon cooling MeTHF solution from 298 to 77 K and gives a similar peak maximum at 649 nm, with a narrower emission profile at 77 K and a shoulder at the lower wavelength (Fig. 4).

The PLQY values decrease generally from **M1** to **M8** in all media, with a few exceptions. For instance, in PS matrix complexes **Au3** has a higher PLQY than **Au2**, whereas in toluene solution the PLQY of **Au3** is one order lower than that of **Au2**. The excited state lifetimes of **Au2** are 2–4 times shorter than for **Au3**. This behavior is likely a reflection of the relative rigidity of the tribenzoazepine ligand (Z = *o*-phenylene) compared with the rather flexible 10,11-dihydrobenzazepine ring (Z = ethylene, *vide supra*). The gold complexes **Au1–Au6** show short excited state lifetimes of the order of 0.2–2.8 μs in PS and 0.1–0.7 μs in toluene at 298 K, leading to radiative rate constants *k*_r_ of 10^4^ to 10^6^ s^–1^. The non radiative rate constants *k*_*n*r_ increase up to 40 times from **M1** to **M4/6** in line with the decreasing band gaps, which is consistent with the energy gap law.^42^

Copper complexes are generally less emissive than their gold analogues, with the highest PLQY being displayed by **Cu1** (49.5% in toluene). The lower PLQY values for the copper complexes correlates well with the lower oscillator strength coefficients for S_1_ → S_0_ transition and smaller contribution of the metal orbitals into LUMO compared with the analogous gold complexes (see ESI, Tables S3 and S5†). The copper analogues show excited state lifetime values that are up to three times longer than for the gold analogs (Table 2). The differences due to ligand rigidity are even more pronounced: **Cu3**, with flexible ethylene bridge, is almost non-emissive (PLQY 2%), while the emission of **Cu2** is 18 times more intense due to rigid *o*-phenylene bridge.

**Fig. 5:**
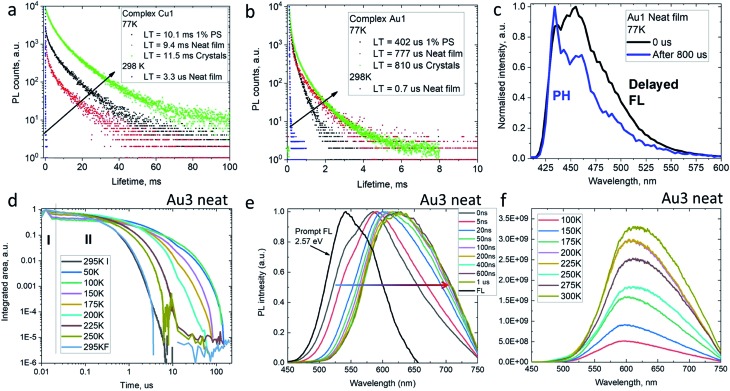
Emission decay kinetics for complexes **Cu1** (a) and **Au1** (b) at 298 and 77 K in the solid state with values for the slow decay; (c) transient emission photoluminescence for **Au1** as neat film (excitation at 380 nm); (d) temperature-dependent time-resolved PL for **Au3** as neat film. (e) Prompt (0–1 ns) and delayed (up to 1 μs delay) emission spectra at 295 K; (f) varied-temperature PL of **Au3** in a neat film in microsecond regime (**II**).

The decay kinetics for complexes **M1** on cooling from 298 to 77 K show an increase in excited state lifetimes of three orders of magnitude in the solid state, *e.g.* from 3.3 μs to 9.4 ms for **Cu1** (Fig. 5), suggesting that PL at room temperature is due to a thermally activated process. Unlike the behavior in solution, the PL profiles of solid samples of **Cu1** and **Au1** at 77 K show a marginal blue-shift with unresolved vibronic structure, indicative of a mixed PL from both ^1^CT and ^3^LE states. In agreement with this, the data are best fitted by a biexponential model. Time-resolved emission spectroscopy and applying a delay of 800 μs confirmed the presence of a ^3^LE emission with characteristic vibronic structure from the sulfone ligand (see for example Fig. 5c).

To get insight into the activation parameters for flexible amide complexes, we have measured the temperature-dependent transient PL for the bright complex **Au3** in neat films. All PL transients show non-exponential kinetics over two distinct time regimes – nanosecond (I) and microsecond (II) as shown on Fig. 5d. The nanosecond regime (I) exhibits no significant *T* dependence, with a lifetime of *ca.* 3 ns limited by instrument response. Spectral deconvolution allowed extraction of the emission profile for the prompt fluorescence, which is blue-shifted compared to the delayed emission at later times (Fig. 5e). The estimated energy of the prompt fluorescence is in excess of 2.57 eV. The microsecond regime (II) shows unstructured and thermally activated luminescence with an activation energy of 94 meV (Fig. S12†). The excited state lifetime increases significantly on cooling, from *τ*_II_ = 800 ns at 300 K to 52 μs below 100 K. We measured photoluminescence intensity as a function of temperature to determine the nature of the thermally activated emission process for complex **Au3** and discriminate *vs.* rigidity effects which could also lead to longer excited state lifetime at low temperature. The photoluminescence intensity increases upon warming from 100 to 300 K (Fig. 5f) thus indicating that it's primarily the radiative rate that is thermally activated and increases with temperature. We observe a red-shift of the delayed emission upon warming for **Au3** (Fig. 5f), which is similar to the behavior of the rigid carbazolate systems.^15^,^29^ The delayed regime dominates the total emission at room temperature, and even on cooling to below 100 K the phosphorescence spectrum could not be resolved. This prevents identification of the ^3^LE energy level even after applying long delay up to 200 μs. Similar behavior has been noted before for other carbene metal amides.^15^,^29^,^31^

### Mechanochromic properties

**Fig. 6:**
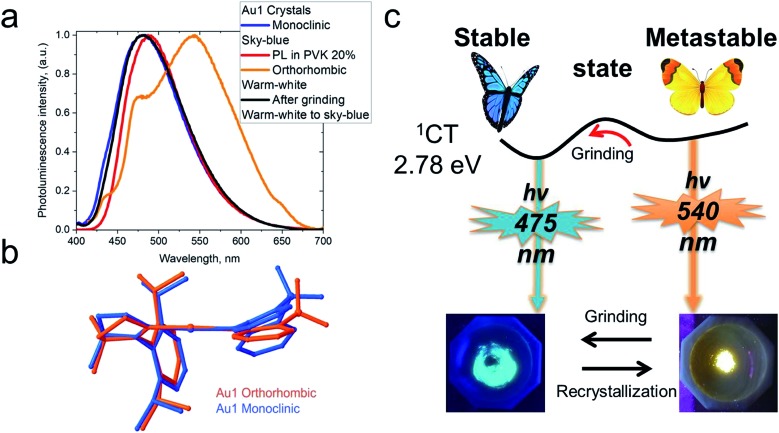
(a) Solid-state PL of crystals of **Au1** in the monoclinic and orthorhombic phases; (b) superposition of the geometries of the **Au1** phases determined by single crystal X-ray diffraction; (c) schematic diagram for the interconversion between stable sky-blue (monoclinic) and warm-white (orthorhombic) emissive forms.

CMA complexes bearing rigid carbazolate ligands are known for their triboluminescence and mechanochromic photoluminescence.^2^,^15^ This behavior may be expected to be even more pronounced in complexes with flexible amide ligands. Indeed, **Au1** shows a mechanochromic response due to its ability to crystallize in two different modifications (*vide supra*): monoclinic crystals show a bright featureless sky-blue emission at 475 nm, whereas the orthorhombic form emits warm-white light with a maximum at 540 nm accompanied by a minor peak at 475 nm (30% PLQY) (Fig. 6). Both modifications differ slightly in the folding angle of the amide ligand; the behavior of **Au1** is therefore likely the result of the different ground state geometries associated with a different angles *α*/*β*, as shown in Fig. 6. The orthorhombic crystals are very fragile and emit sky-blue light if broken, which explains the origin of the high-energy peak in the warm-white emission profile. Grinding the orthorhombic crystals leads to a hypsochromic shift of 0.32 eV and the disappearance of the broad low energy emission, while the new sky-blue emission almost overlaps with the PL of the monoclinic form. The additional emission at 475 nm for the orthorhombic crystals is therefore due to the mechanoresponsive behavior of this structure. The mechanoresponse of **Au1** is irreversible, that is the warm-white emission can only be obtained after recrystallization, and the effect is therefore most probably due to crystal packing. The switch between the stable blue CT and metastable warm white emission can however be exploited for the fabrication of white emissive OLEDs (*vide infra*).

### Electroluminescent (EL) properties

**Fig. 7:**
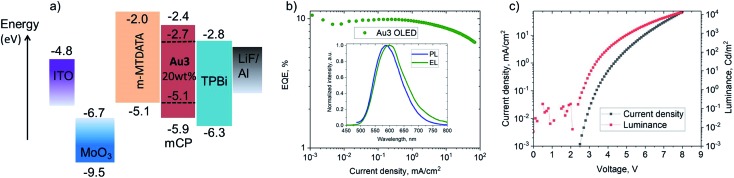
(a) Vapour-deposited OLED device architecture based on **Au3**; (b) external quantum efficiencies *vs.* current density of champion OLEDs with inset showing the electroluminescence (EL) and PL spectra; (c) current density/luminance *vs.* voltage curve of champion OLEDs.

Vapor-deposited OLEDs incorporating **Au3** as emitter were fabricated with the architecture shown in Fig. 7. OLEDs with 20 wt% dopant in 1,3-bis(9-carbazolyl)benzene (mCP) show EL peaks at *λ*_em_ = 590 nm (Fig. 7), while the EL spectra closely match the microsecond-regime PL spectra. Fig. 7 shows the current density–voltage and luminance–voltage characteristics of evaporated devices. External quantum efficiencies (EQEs) (for **Au3** of 9.8% at 100 cd m^–2^ and 9.1% at 1000 cd m^–2^) were achieved, with low roll-off characteristics. The low turn-on voltage of only 2.6 V indicates a good charge balance in OLED device (Table 3).

**Table 3:** Performance data of solution and evaporated OLEDs

Dopant [wt%]/method	*V*_ON_ ^*b*^ [V]	*η*_EQE,EL_ [%] (max.)	*η*_EQE,EL_ [%] (100 cd m^–2^)	*η*_EQE,EL_ [%] (1000 cd m^–2^)	CIE^*a*^ (*x*, *y*)
**Au1**, [20%] solution, PVK	5.4	5.8	5.5	3.6	(0.15, 0.24)
**Au1**, [20%] solution, CBP	5.3	4.6	4.2	2.3	(0.18, 0.31)
**Au3**, [20%] evaporated, mCP	2.6	11.0	9.8	9.1	(0.53, 0.46)

^*a*^Commission Internationale de l'Éclairage (CIE) color co-ordinates.

^*b*^ *V*_ON_ determined at a luminance of 1 cd m^–2^.

**Fig. 8:**
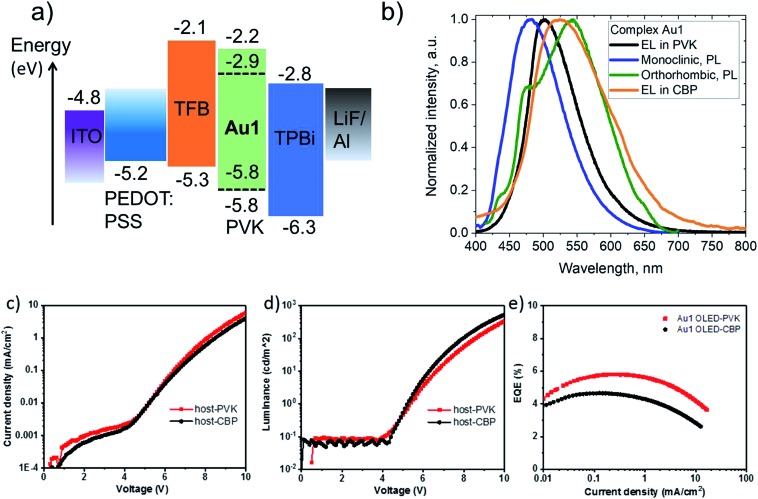
(a) Solution-processed **Au1**-based OLED architectures based on 20 wt% doped PVK as the emissive layer (EML); (b) photoluminescence (PL) and electroluminescence (EL) spectra of different conformers and matrices; (c) current–voltage characteristics and (d) luminance–voltage characteristics for **Au1**-OLED; (e) external quantum efficiencies *vs.* current density.

Complex **Au1** proved insufficiently stable for thermal vapor deposition but suitable for the fabrication of solution-processed OLEDs, with the device architecture ITO/PEDOT:PSS (40 nm)/TFB (10 nm)/PVK or CBP with 20%wt **Au1** (40 nm)/TPBi (70 nm)/LiF (0.8 nm)/Al (100 nm). The OLED glowed initially sky-blue, but in the PVK host the emission quickly (<1 s) broadened and changed to warm-white EL. This transition is likely connected with existence of the two conformational isomers isolated in the crystals of **Au1**, *vide supra* (Fig. 6). The initial sky-blue EL is tentatively attributed to an emission from the monoclinic form, while the final warm-white EL largely agrees with the averaged PL profiles of the monoclinic and orthorhombic forms (Fig. 8). This explanation was further supported by a second set of **Au1** OLEDs in CBP as host, where the EL profile largely coincides with the warm-white PL of the orthorhombic **Au1** form. The devices produced peak EQEs of 5.8% and 4.6% for **Au1** in PVK and CBP hosts, respectively. This is almost the maximum that can be achieved considering the 30% PLQY value for the warm-white orthorhombic form and 39% PLQY value in 20% PVK (Fig. 8).

## Conclusion

Carbene metal amide (CMA) complexes of copper and gold of the type (L)M-N(C_6_H_4_)_2_Z, where Z = O, S, NMe, CMe_2_, SO_2_, C_2_H_4_ or *o*-C_6_H_4_ provide a range of photoemissive complexes in which the HOMO–LUMO gap is determined by the electronic characteristics of Z, to give photoluminescence ranging from blue to deep red. The amide-N atoms are part of non-planar, flexible 6- and 7-membered heterocycles (“butterfly” conformation). These conformational changes influence the emission behavior. Similar to previously reported CMA compounds with rigid carbazole ligands,^13^–^15^,^29^–^32^ the complexes described here emit predominantly *via* a thermally activated emission process with sub-microsecond to microsecond excited state lifetimes at room temperature, with radiative rates exceeding 10^6^ s^–1^. Nitro-substituents on the amide ligands, on the other hand, quench L(M)LCT processes and luminesce. On cooling to 77 K the emissions are blue-shifted and excited state lifetimes increase by three orders of magnitude. **M1** (M = Cu and Au) complexes emit *via* both delayed fluorescence and phosphorescence at 77 K whereas **Au3** shows predominantly delayed fluorescence. The magnitude of *k*_nr_ increases across the gold and copper series in line with the emission wavelengths, which is consistent with the energy gap law. Proof-of-concept OLEDs were fabricated by thermal vapor deposition, with EQEs of up to 9.8% at 100 cd m^–2^ for a yellow emitter based on the **Au3** complex with the 7-ring amide ligand. The gold complex **Au1** can adopt two conformations which show either blue or warm-white PL; this behavior was exploited in a solution-processed OLED based on **Au1** to provide the first example of a CMA-based white-emitting OLED. Conformational flexibility can therefore be employed as a useful design feature to tailor the emission energies and increase the range of readily accessible CMA materials for light-emitting applications.

## Conflicts of interest

The authors declare no competing financial interest.

## Supplementary Material

Supplementary information

Crystal structure data
