# Supplementary material for: Carbene metal amide photoemitters: tailoring conformationally flexible amides for full color range emissions including white-emitting OLED
Source: Chem Sci. 2019 Nov 13;11(2):435–46. doi: 10.1039/c9sc04589a (PMC7067249; doi:10.1039/c9sc04589a)
Supplement: Supplementary file 1 [file SC-011-C9SC04589A-s001.pdf]

Electronic Supplementary Material (ESI) for Chemical Science.
This journal is © The Royal Society of Chemistry 2019

Supporting Information

Synthetic procedures p. S1
X-ray Crystallography p. S11
Electrochemistry p. S17
Photophysical characterisation p. S24
Computational details p. S28
OLED device fabrication p. S44

Table S1 Redox data for copper and gold complexes. p. S21
Table S2. Ground and excited state optimized structures for Cu, Au complexes: p. S28
Table S3. HOMOs and LUMOs of CMA complexes p. S32
Table S4. HOMO/LUMO gap, overlap integrals, HOMO-LUMO distances p. S37
Table S5. S_1 / T_1 energies, vertical excitation character, $S_0 \rightarrow S_1$ oscillator strength p. S37
Table S6. Dipole moments in S0 and S1 states in S0 geometry p. S41

EXPERIMENTAL SECTION

General Considerations. Unless stated otherwise all reactions were carried out in air. Solvents were distilled and dried as required. Sodium *tert*-butoxide, oxazine, phenothiazine, 9H-tribenzo[b,d,f]azepine were purchased from Sigma-Aldrich and used as received. The organic ligands (^{Ad}L),[1] 10H-phenothiazine 5,5-dioxide and complexes (^{Ad}L)MCl (M = Cu and Au),[2] were obtained according to literature procedures. ^{1}H, ^{13}C{^{1}H} and ^{19}F NMR spectra were recorded using a Bruker Avance DPX-300 MHz NMR spectrometer. ^{1}H NMR spectra (300.13 MHz) and ^{13}C{^{1}H} (75.47 MHz) were referenced to CD_2Cl_2 at δ 5.32 (^{13}C, δ 53.84), C_6D_6 at δ 7.16 (^{13}C, δ 128.4), $CDCl_3$ at δ 7.26 (^{13}C, δ 77.16). IR spectra were recorded using a Perkin-Elmer Spectrum One FT-IR spectrometer equipped with a diamond ATR attachment. All electrochemical experiments were performed using an Autolab PGSTAT 302N computer-controlled potentiostat. Cyclic voltammetry (CV) was performed using a three-electrode configuration consisting of either a glassy carbon macrodisk working electrode (GCE) (diameter of 3 mm; BASi, Indiana, USA) combined with a Pt wire counter electrode (99.99 %; GoodFellow, Cambridge, UK) and an Ag wire pseudoreference electrode (99.99 %; GoodFellow, Cambridge, UK). The GCE was polished between experiments using alumina slurry (0.3 μm), rinsed in distilled water and subjected to brief sonication to remove

any adhering alumina microparticles. The metal electrodes were then dried in an oven at 100 °C to remove residual traces of water, the GCE was left to air dry and residual traces of water were removed under vacuum. The Ag wire pseudoreference electrodes were calibrated to the ferrocene/ferrocenium couple in MeCN at the end of each run to allow for any drift in potential, following IUPAC recommendations.[3] All electrochemical measurements were performed at ambient temperatures under an inert Ar atmosphere in MeCN containing complex under study (0.14 mM) and supporting electrolyte [n-Bu_4N][PF_6] (0.13 mM). Data were recorded with Autolab NOVA software (v. 1.11). Elemental analyses were performed by London Metropolitan University.

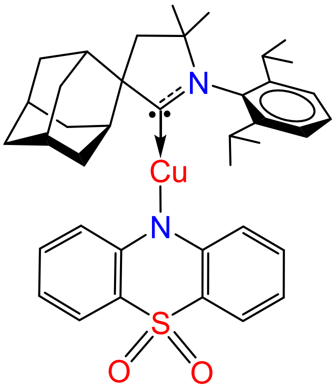

Synthesis of (^{Ad}CAAC)Cu(phenothiazin-10-ide 5,5-dioxide) (Cu1) A mixture of (^{Ad}L)CuCl (0.4 g, 0.84 mmol), NaO^{t}Bu (81 mg, 0.84 mmol) and 10H-phenothiazine 5,5-dioxide (0.194 g, 0.84 mmol) in dry THF (60 mL) under an argon atmosphere was stirred for 8 h. The mixture was filtered through the 3 cm pad of Celite. All volatiles were evaporated and residue was crystallized by layering saturated toluene solution with hexane to give off-white solid. Product was dried under vacuum at 140 °C for 3h. Yield: 0.508 g (0.76 mmol, 90 %).

^{1}H NMR (300 MHz, CD_2Cl_2): δ 7.77 (dd, J = 7.9 and 1.4 Hz, 2H, amide CH^4), 7.62 (t, J = 7.8 Hz, 1H, aryl), 7.41 (d, J = 7.8 Hz, 2H, aryl), 7.07 (td, J = 7.9 and 1.4 Hz, 2H, amide CH^3), 6.85 (t, J = 7.4 Hz, 2H, CH^2), 6.35 (d, J = 8.4 Hz, 2H, CH^1), 3.47 (d, J = 12.4 Hz, 2H, adamantyl CH_2), 2.91 (sept, J = 6.8 Hz, 2H, C*H*Me_2), 2.30–1.81 (m, 14H, adamantyl CH and CH_2), 1.40 (s, 6H, C($C\underline{H}_3$)), 1.33 (d, J = 6.8 Hz, 6H, CHMe_2) overlapping with 1.18 (d, J = 6.8 Hz, 6H, CHMe_2). ^{13}C NMR (75 MHz, CD_2Cl_2) δ 253.1 (C carbene), 148.7 (*ipso*-CN amide), 145.6 (*o*-C), 136.2 (*ipso*-C), 131.3 (amide CH^3), 130.4 (*p*-CH), 125.8 (*m*-CH), 123.6 (amide CH^1), 122.2 (amide CH^4), 122.1 (*ipso*-CS amide), 118.3 (amide CH^2), 79.4 (C_q), 65.5

(C_q), 48.1 (CH_2), 38.7 (CH_2), 37.5 (CH), 36.1 (CH_2), 34.4 (CH_2), 29.6, 29.5, 28.2, 27.4, 26.6, 22.9 (CH_3). Anal. Calcd. for $C_{39}H_{47}CuN_2O_2S$ (670.27): C, 69.77; H, 7.06; N, 4.17. Found: C, 69.45; H, 6.84; N, 4.10.

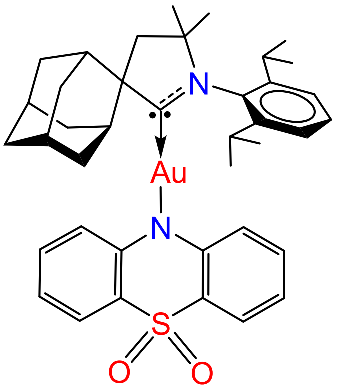

Synthesis of (^{Ad}CAAC)Au(phenothiazin-10-ide 5,5-dioxide) (Au1) Following the procedure described for **1**, the complex was made from (^{Ad}L)AuCl (0.35 g, 0.57 mmol), NaO^{t}Bu (55 mg, 0.57 mmol) and 10H-phenothiazine 5,5-dioxide (0.133 g, 0.57 mmol) as a white solid. Yield: 0.405 g (0.5 mmol, 88 %).

^{1}H NMR (300 MHz, CD_2Cl_2): δ 7.82 (d, J = 7.6 Hz, 2H, amide CH^4), 7.66 (t, J = 8.0 Hz, 1H, aryl), 7.42 (d, J = 8.0 Hz, 2H, aryl), 7.13 (t, J = 7.2 Hz, 2H, amide CH^3), 6.85–6.92 (m, 4H, overlapping amide CH^1 and CH^2), 4.04 (d, J = 12.7 Hz, 2H, adamantyl CH_2), 2.85 (sept, J = 6.4 Hz, 2H, C*H*Me$_2$), 2.43–1.81 (m, 14H, adamantyl CH and CH_2), 1.42 (s, 6H, C($C\underline{H}_3$)), 1.32 (d, J = 6.6 Hz, 6H, CH*Me*$_2$) overlapping with 1.30 (d, J = 6.6 Hz, 6H, CH*Me*$_2$). ^{13}C NMR (75 MHz, CD_2Cl_2) δ 240.9 (C carbene), 148.0 (*ipso*-CN amide), 145.8 (*o*-C), 136.1 (*ipso*-C), 131.2 (amide CH^3), 130.2 (*p*-CH), 125.8 (*m*-CH), 122.9 (amide CH^1), 122.4 (*ipso*-CS amide), 122.3 (amide CH^4), 118.9 (amide CH^2), 77.8 (C_q), 64.6 (C_q), 48.8 (CH_2), 39.2 (CH_2), 37.4 (CH), 35.3 (CH_2), 34.7 (CH_2), 29.5, 29.4, 28.2, 27.5, 26.4, 23.3 (CH_3). Anal. Calcd. for $C_{39}H_{47}AuN_2O_2S$ (804.30): C, 58.20; H, 5.89; N, 3.48. Found: C, 58.55; H, 6.13; N, 3.39.

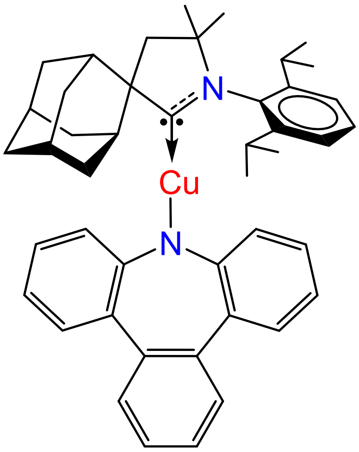

Synthesis of (^{Ad}CAAC)Cu(tribenzo[b,d,f]azepin-9-ide) (Cu2). Following the procedure described for **1**, the complex was made from (^{Ad}CAAC)CuCl (0.2 g, 0.42 mmol), NaO^{t}Bu (40 mg, 0.42 mmol) and 9H-tribenzo[b,d,f]azepine (102 mg, 0.42 mmol) as a yellow powder. Yield: 0.246 g (0.36 mmol, 86 %).

^{1}H NMR (300 MHz, CD_2Cl_2): δ 7.48–7.42 (m, 1H aryl, 4H benzo[f] and 2H amide CH^4), 7.29 (d, J = 7.7 Hz, 2H, aryl), 7.23 (t, J = 7.5 Hz, 2H, amide CH^3), 7.12 (t, J = 7.5 Hz, 2H, amide CH^2), 6.94 (d, J = 7.7 Hz, 2H, amide CH^1), 3.58 (d, J = 12.0 Hz, 2H, adamantly CH_2), 2.80 (sept, J = 6.6 Hz, 2H, C*H*Me$_2$), 2.21–1.77 (m, 14H, adamantyl CH and CH_2), 1.31 (s, 6H, C(C$\underline{H}_3$)) overlapping with 1.29 (d, J = 6.6 Hz, 12H, CH*Me*$_2$). ^{13}C NMR (75 MHz, CD_2Cl_2) δ 252.5 (C carbene), 151.5 (*ipso*-CN amide), 145.4 (*o*-C, Dipp), 139.8 (*ipso*-C amide) 135.8 (*ipso*-C, Dipp), 132.8 (*ipso*-C amide), 130.4 (amide CH), 130.3 (amide CH), 129.8 (*p*-CH, Dipp), 128.9 (amide CH), 128.1 (amide CH), 125.0 (*m*-CH), 124.4 (amide CH), 120.1 (amide CH), 78.8 (C_q), 64.9 (C_q), 48.2 (CH_2), 39.0 (CH_2), 37.5 (CH), 36.0 (CH_2), 34.5 (CH_2), 29.5, 29.4, 28.3, 27.6, 26.9, 22.6 (CH_3). Anal. Calcd. for $C_{45}H_{51}N_2Cu$ (682.33): C, 79.08; H, 7.52; N, 4.10. Found: C, 79.34; H, 7.71; N, 4.03.

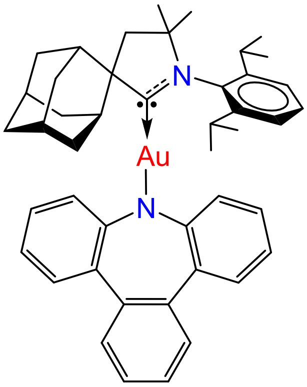

Synthesis of (^{Ad}CAAC)Au(tribenzo[b,d,f]azepin-9-ide) (Au2). Following the procedure described for **1**, the complex was made from (^{Ad}CAAC)AuCl (0.4 g, 0.66 mmol), NaO^{t}Bu (66 mg, 0.66 mmol) and 9H-tribenzo[b,d,f]azepine (160 mg, 0.66 mmol) as a yellow powder. The product was crystallized by layering a saturated toluene solution with hexane.Yield: 0.324 g (0.4 mmol, 60 %).

^{1}H NMR (300 MHz, CD_2Cl_2): δ 7.50 (t, J = 7.7 Hz, 1H, aryl), 7,27 (d, J = 7.7 Hz, 2H, aryl) overlapping with 7.22–7.24 (m, 2H, amide), 7.11–7.15 (m, 4H, amide), 6.69–6.79 (m, 4H, amide), 6.41 (d, J = 7.5 Hz, 2H, amide CH^1), 3.99 (d, J = 12.5 Hz, 2H, adamantly CH_2), 2.83 (sept, J = 6.6 Hz, 2H, C*H*Me$_2$), 2.34–1.75 (m, 14H, adamantyl CH and CH_2), 1.35 (s, 6H, C(C$\underline{H}_3$)) overlapping with 1.32 (d, J = 6.6 Hz, 6H, CH*Me*$_2$) and 1.28 (d, J = 6.6 Hz, 6H,

CH*Me*$_2$). ^{13}C NMR (75 MHz, CD_2Cl_2) δ 243.5 (C carbene), 162.9 (*ipso*-C amide), 151.5 (*ipso*-CN amide), 145.6 (*o*-C, Dipp), 143.2 (*ipso*-C amide) 136.0 (*ipso*-C, Dipp), 130.1 (amide CH), 129.7 (*p*-CH, Dipp), 129.6 (amide CH), 127.5 (amide CH), 127.0 (amide CH), 126.0 (amide CH), 125.4 (*m*-CH), 120.8 (amide CH), 76.6 (C_q), 64.2 (C_q), 48.8 (CH_2), 39.2 (CH_2), 37.2 (CH), 35.5 (CH_2), 34.8 (CH_2), 29.4, 29.3, 28.1, 27.7, 26.2, 23.4 (CH_3). Anal. Calcd. for $C_{45}H_{51}N_2Au$ (816.37): C, 66.17; H, 6.29; N, 3.43. Found: C, 66.35; H, 6.38; N, 3.50.

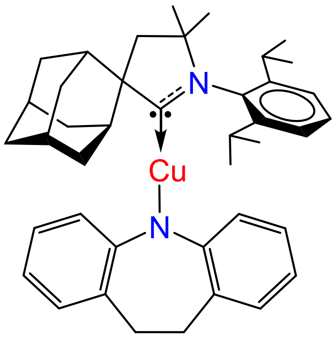

Synthesis of (^{Ad}CAAC)Cu(10,11-dihydrodibenz[b,f]azepin-5-ide) (Cu3). Following the procedure described for **1**, the complex was made from (^{Ad}CAAC)CuCl (0.2 g, 0.42 mmol), NaO^{t}Bu (40 mg, 0.42 mmol) and 10,11-dihydro-5H-dibenz[b,f]azepine (82 mg, 0.42 mmol) as an orange powder. Yield: 0.209 g (0.33 mmol, 78 %).

^{1}H NMR (300 MHz, CD_2Cl_2): δ 7.45 (t, *J* = 7.6 Hz, 1H, aryl), 7.29 (d, *J* = 7.6 Hz, 2H, aryl), 7.07–7.01 (m, 4H, azepine), 6.77–6.72 (m, 4H, azepine), 3.57 (d, *J* = 13.2 Hz, 2H, CH_2), 3.05 (s, 4H, CH_2 azepine), 2.80 (sept, *J* = 6.6 Hz, 2H, C*H*Me$_2$), 2.22–1.77 (m, 14H, adamantyl CH and CH_2), 1.32 (s, 6H, C($\underline{H}_3$)), 1.29 (d, *J* = 6.6 Hz, 12H, CH*Me*$_2$). ^{13}C NMR (75 MHz, CD_2Cl_2) δ 252.9 (C carbene), 145.0 (*o*-C), 142.5 (*ipso*-CN azepine), 135.4 (*ipso*-C), 130.5 (azepine CH), 129.4 (*p*-CH), 128.6 (azepine *ipso*-C), 126.7 (*m*-CH), 124.7 (azepine CH), 119.2 (azepine CH), 117.8 (azepine CH), 78.4 (C_q), 64.5 (C_q), 47.8 (CH_2), 38.6 (CH_2), 37.1 (CH), 35.7 (CH_2), 34.9 (CH_2), 34.1 (azepine CH_2), 29.1, 29.0, 28.0, 27.2, 26.6, 22.2 (CH_3). Anal. Calcd. for $C_{41}H_{51}N_2Cu$ (635.40): C, 77.50; H, 8.09; N, 4.41. Found: C, 77.62; H, 8.16; N, 4.50.

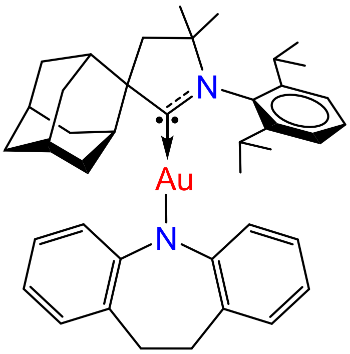

Synthesis of (^{Ad}CAAC)Au(10,11-dihydrodibenz[b,f]azepin-5-ide) (Au3). Following the procedure described for **1**, the complex was made from (^{Ad}CAAC)AuCl (0.2 g, 0.33 mmol), NaO^{t}Bu (33 mg, 0.33 mmol) and 10,11-dihydro-5H-dibenz[b,f]azepine (64.3 mg, 0.33 mmol) as an orange powder. Yield: 0.169 g (0.22 mmol, 66 %).

^{1}H NMR (300 MHz, CD_2Cl_2): δ 7.53 (t, J = 7.6 Hz, 1H, aryl), 7.30 (d, J = 7.6 Hz, 2H, aryl), 6.75 (d, J = 7.4 Hz, 2H, azepine CH^4), 6.69 (d, J = 7.4 Hz, 2H, azepine CH^1), 6.62 (t, J = 7.4 Hz, 2H, azepine CH^3), 6.43 (t, J = 7.4 Hz, 2H, azepine CH^2), 3.95 (d, J = 13.2 Hz, 2H, CH_2), 2.89 (s, 4H, CH_2 azepine), 2.83 (sept, J = 6.6 Hz, 2H, C*H*Me$_2$), 2.14–1.69 (m, 14H, adamantyl CH and CH_2), 1.36 (s, 6H, C(C$\underline{H}_3$)), 1.34 (d, J = 6.6 Hz, 6H, CH*Me*$_2$), 1.30 (d, J = 6.6 Hz, 6H, CH*Me*$_2$). ^{13}C NMR (75 MHz, CD_2Cl_2) δ 242.3 (C carbene), 153.9 (*ipso*-CN azepine), 145.4 (*o*-C), 136.4 (*ipso*-C), 129.8 (*p*-CH), 129.5 (azepine CH^4), 127.0 (azepine *ipso*-C), 125.7 (azepine CH^3), 125.4 (*m*-CH), 124.5 (azepine CH^1), 116.7 (azepine CH^2), 76.6 (C_q), 64.1 (C_q), 48.8 (CH_2), 39.3 (CH_2), 37.2 (CH), 36.4 (CH_2), 35.0 (CH_2), 34.8 (azepine CH_2), 29.3, 28.0, 27.6, 26.0, 23.4 (CH_3). Anal. Calcd. for $C_{41}H_{51}N_2Au$ (768.82): C, 64.05; H, 6.69; N, 3.64. Found: C, 64.27; H, 6.83; N, 3.51.

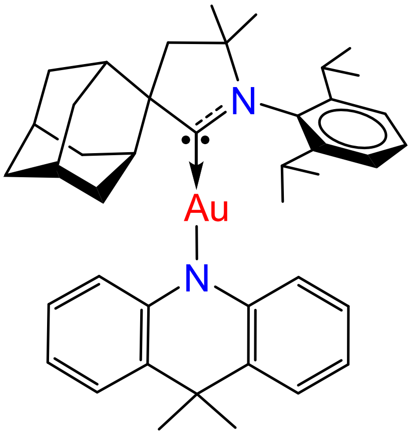

Synthesis of (^{Ad}CAAC)Au(9,9-dimethylacridin-10(9H)-yl) (Au4). Following the procedure described for **1**, the complex was made from (^{Ad}CAAC)AuCl (0.2 g, 0.33 mmol),

NaO^{t}Bu (33 mg, 0.33 mmol) and 9,10-dihydro-9,9-dimethylacridine (69 mg, 0.33 mmol) as a red powder. Yield: 0.240 g (0.307 mmol, 93 %).

^{1}H NMR (300 MHz, CD_2Cl_2): δ 7.60 (t, *J* = 7.8 Hz, 1H, aryl), 7.37 (d, *J* = 7.8 Hz, 2H, aryl), 7.12 (d, *J* = 7.5 Hz, 2H, CH^4), 6.67 (t, *J* = 7.5 Hz, 2H, CH^3), 6.51 (t, *J* = 7.3 Hz, 2H, CH^2), 6.44 (t, *J* = 8.0 Hz, 2H, CH^2), 4.15 (d, *J* = 12.3 Hz, 2H, CH_2), 2.87 (sept, *J* = 6.6 Hz, 2H, C*H*Me_2), 2.40–1.82 (m, 14H, adamantyl CH and CH_2), 1.39 (s, 6H, C($C\underline{H}_3$) CAAC), 1.36 (s, 6H, C($C\underline{H}_3$) amide) overlapping with 1.34 (d, *J* = 6.6 Hz, 6H, CH*Me*$_2$) overlapping with 1.31 (d, *J* = 6.6 Hz, 6H, CH*Me*$_2$). ^{13}C NMR (75 MHz, CD_2Cl_2) δ 243.1 (C carbene), 148.9 (*ipso*-C amide), 145.7 (*o*-C), 136.2 (*ipso*-C), 131.2 (*ipso*-C amide), 129.9 (*p*-CH), 125.6 (*m*-CH overlapping with amide CH^3), 124.4 (amide CH^4), 118.3 (amide *C*H^1), 117.1 (amide *C*H^2), 77.1 (C_q), 64.5 (C_q), 49.0 (CH_2), 39.4 (CH_2), 37.6 (CH), 35.2 (CH_2), 34.8 (CH_2), 30.5 (*ipso*-$\underline{C}Me_2$ amide,) 29.46, 28.2, 27.7, 26.1, 25.9, 23.4 (CH_3). Anal. Calcd. for $C_{42}H_{35}N_2Au$ (782.87): C, 64.44; H, 6.82; N, 3.58. Found: C, 64.68; H, 7.04; N, 3.46.

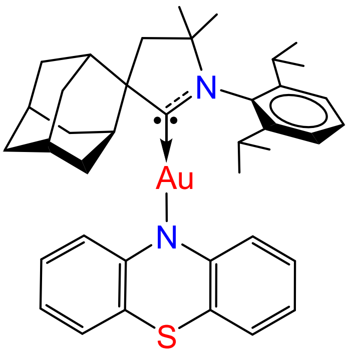

Synthesis of (^{Ad}CAAC)Au(phenothiazine) (Au5). Following the procedure described for **1**, the complex was made from (^{Ad}CAAC)AuCl (0.3 g, 0.49 mmol), NaO^{t}Bu (48 mg, 0.49 mmol) and phenothiazine (97.5 mg, 0.49 mmol) as an orange powder. Yield: 0.347 g (0.45 mmol, 91 %).

^{1}H NMR (300 MHz, CD_2Cl_2): δ 7.56 (t, *J* = 7.8 Hz, 1H, aryl), 7.34 (d, *J* = 7.8 Hz, 2H, aryl), 6.60 (dd, *J* = 7.4 and 1.3 Hz, 2H, amide CH^4), 6.55 (td, *J* = 7.4 and 1.3 Hz, 2H, amide CH^3), 6.41 (td, *J* = 7.4 and 1.3 Hz, 2H, amide CH^2), 6.13 (d, *J* = 7.8 Hz, 2H, amide CH^1), 4.01 (d, *J* = 12.0 Hz, 2H, adamantyl CH_2), 2.85 (sept, *J* = 6.7 Hz, 2H, C*H*Me_2), 2.39–1.82 (m, 14H, adamantyl CH and CH_2), 1.39 (s, 6H, C($C\underline{H}_3$)), 1.35 (d, *J* = 6.7 Hz, 6H, CH*Me*$_2$), 1.31 (d, *J* = 6.7 Hz, 6H, CH*Me*$_2$). ^{13}C NMR (75 MHz, CD_2Cl_2) δ 242.5 (C carbene), 152.5 (*ipso*-CN amide), 145.7 (*o*-C), 136.3 (*ipso*-C), 129.8 (*p*-CH), 126.7 (amide CH^3), 126.1 (amide CH^4), 125.6 (*m*-CH), 120.8 (*ipso*-CS amide), 119.4 (amide CH^2), 118.8 (amide CH^1), 77.1 (C_q), 64.4 (C_q), 48.8 (CH_2), 39.3 (CH_2), 37.3 (CH), 35.2 (CH_2), 34.7 (CH_2), 29.4, 29.3, 28.1, 27.6,

26.3, 23.4 (CH_3). Anal. Calcd. for $C_{39}H_{47}N_2AuS$ (772.31): C, 60.61; H, 6.13; N, 3.62. Found: C, 60.96; H, 6.23; N, 3.70.

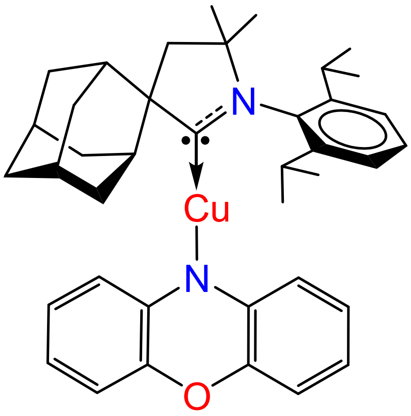

Synthesis of (^{Ad}CAAC)Cu(oxazine) (Cu6). Following the procedure described for **1**, the complex was made from (^{Ad}CAAC)CuCl (0.2 g, 0.42 mmol), NaO^{t}Bu (40 mg, 0.42 mmol) and oxazine (77 mg, 0.42 mmol) as an orange powder. Yield: 0.248 g (0.40 mmol, 95 %).
^{1}H NMR (300 MHz, CD_2Cl_2): δ 7.54 (t, *J* = 7.9 Hz, 1H, aryl), 7.35 (d, *J* = 7.9 Hz, 2H, aryl), 6.20 (t, *J* = 7.6 Hz, 2H, oxazine CH^2), 6.15–6.06 (m, 4H, oxazine CH^3 and CH^4), 5.23 (d, *J* = 7.6 Hz, 2H, oxazine CH^1), 3.46 (d, *J* = 12.8 Hz, 2H, CH_2), 2.86 (sept, *J* = 6.6 Hz, 2H, C*H*Me$_2$), 2.26–1.80 (m, 14H, adamantyl CH and CH_2), 1.35 (s, 6H, C($C\underline{H}_3$)), 1.31 (d, *J* = 6.6 Hz, 6H, CH*Me*$_2$), 1.22 (d, *J* = 6.6 Hz, 6H, CH*Me*$_2$). ^{13}C NMR (75 MHz, CD_2Cl_2) δ 252.8 (C carbene), 146.1 (*ipso*-CN oxazine), 145.2 (*o*-C), 143.8 (*ipso*-CO oxazine), 135.6 (*ipso*-C), 129.6 (*p*-CH), 125.1 (*m*-CH), 123.0 (oxazine CH^2), 117.0 (oxazine CH^1), 116.3 (oxazine CH), 113.4 (oxazine CH), 78.4 (C_q), 65.0 (C_q), 48.0 (CH_2), 38.5 (CH_2), 37.2 (CH), 35.5 (CH_2), 34.1 (CH_2), 29.1, 27.9, 27.1, 26.1, 22.4 (CH_3). Anal. Calcd. for $C_{39}H_{47}N_2CuO$ (623.34): C, 75.15; H, 7.60; N, 4.49. Found: C, 75.23; H, 7.65; N, 4.45.

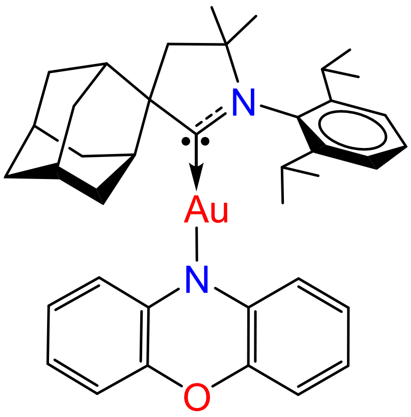

Synthesis of (^{Ad}CAAC)Au(oxazine) (Au6). Following the procedure described for **1**, the complex was made from (^{Ad}CAAC)AuCl (0.2 g, 0.33 mmol), NaO^{t}Bu (33 mg, 0.33 mmol) and oxazine (61.5 mg, 0.33 mmol) as a red powder. Yield: 0.229 g (0.30 mmol, 91 %).

^{1}H NMR (300 MHz, CD_2Cl_2): δ 7.55 (t, *J* = 7.9 Hz, 1H, aryl), 7.34 (d, *J* = 7.9 Hz, 2H, aryl), 6.24 (t, *J* = 7.6 Hz, 2H, oxazine CH^2), 6.19–6.11 (m, 4H, oxazine CH^3 and CH^4), 5.71 (d, *J* = 7.6 Hz, 2H, oxazine CH^1), 4.01 (d, *J* = 12.8 Hz, 2H, CH_2), 2.82 (sept, *J* = 6.6 Hz, 2H, C*H*Me_2), 2.37–1.81 (m, 14H, adamantyl CH and CH_2), 1.36 (s, 6H, C(C$\underline{H}_3$)) overlapping with 1.35 (d, *J* = 6.6 Hz, 6H, CH*Me*$_2$), 1.31 (d, *J* = 6.6 Hz, 6H, CH*Me*$_2$). ^{13}C NMR (75 MHz, CD_2Cl_2) δ 242.3 (C carbene), 146.1 (*ipso*-CN oxazine), 145.7 (*o*-C), 142.8 (*ipso*-CO oxazine), 136.1 (*ipso*-C), 129.7 (*p*-CH), 125.5 (*m*-CH), 122.9 (oxazine CH^2), 117.4 (oxazine CH^1), 116.6 (oxazine CH), 114.0 (oxazine CH), 77.2 (C_q), 64.4 (C_q), 49.0 (CH_2), 39.3 (CH_2), 37.5 (CH), 35.2 (CH_2), 34.7 (CH_2), 29.46, 29.43, 28.2, 27.6, 26.3, 23.3 (CH_3). Anal. Calcd. for $C_{39}H_{47}N_2AuO$ (756.76): C, 61.90; H, 6.26; N, 3.70. Found: C, 62.08; H, 6.35; N, 3.76.

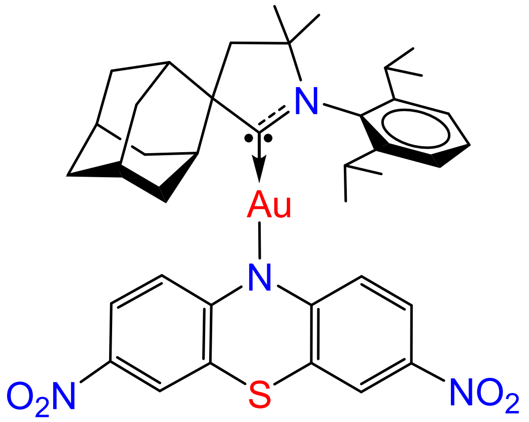

Synthesis of (^{Ad}CAAC)Au(3,7-dinitrophenothiazine) (Au7). Following the procedure described for **1**, the complex was made from (^{Ad}CAAC)AuCl (0.2 g, 0.33 mmol), NaO^{t}Bu (33 mg, 0.33 mmol) and 10-H-3,7-dinitrophenothiazine (95.2 mg, 0.33 mmol) as a dark violet powder. Yield: 0.275 g (0.32 mmol, 97 %).

^{1}H NMR (300 MHz, CD_2Cl_2): δ 7.67 (t, *J* = 7.9 Hz, 1H, aryl), 7.47 (d, *J* = 2.1 Hz, 2H, phenothiazine CH^4), 7.43 (dd, *J* = 9.0 and 2.1 Hz, 2H, phenothiazine CH^2) overlapping with 7.40 (d, *J* = 7.9 Hz, 2H, aryl), 6.15 (d, *J* = 9.0 Hz, 2H, phenothiazine CH^1), 3.85 (d, *J* = 12.8 Hz, 2H, CH_2), 2.82 (sept, *J* = 6.6 Hz, 2H, C*H*Me_2), 2.42–1.81 (m, 14H, adamantyl CH and CH_2), 1.41 (s, 6H, C(C$\underline{H}_3$)), 1.33 (d, *J* = 6.6 Hz, 12H, CH*Me*$_2$). ^{13}C NMR (75 MHz, CD_2Cl_2) δ 240.1 (C carbene), 156.6 (*ipso*-CN phenothiazine), 145.7 (*o*-C), 141.6 (*ipso*-C^3-NO_2 phenothiazine), 136.1 (*ipso*-C), 130.3 (*p*-CH), 125.9 (*m*-CH), 124.1 (phenothiazine CH^2), 121.9 (phenothiazine CH^4), 120.7 (*ipso*-CS phenothiazine), 118.8 (phenothiazine CH^1), 78.0 (C_q), 64.4 (C_q), 48.7 (CH_2), 39.1 (CH_2), 37.4 (CH), 35.3 (CH_2), 34.6 (CH_2), 29.5, 29.4, 28.1,

27.4, 26.4, 23.3 (CH_3). Anal. Calcd. for $C_{39}H_{45}N_4AuSO_4$ (862.82): C, 54.29; H, 5.26; N, 6.49. Found: C, 54.43; H, 5.34; N, 6.52.

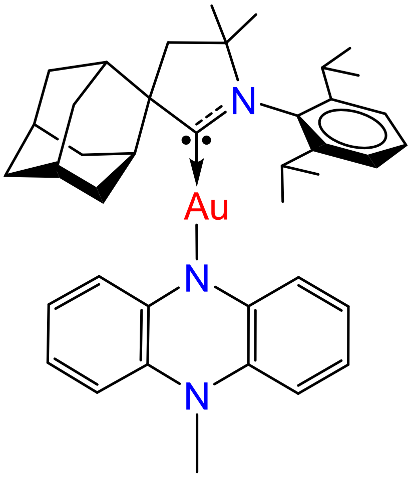

Synthesis of (^{Ad}CAAC)Au(N-methylphenazine) (Au8). Following the procedure described for **1**, the complex was made from (^{Ad}CAAC)AuCl (0.2 g, 0.33 mmol), NaO^{t}Bu (33 mg, 0.33 mmol) and 10-H-N-methylphenazine (65 mg, 0.33 mmol) as a deep violet powder. Yield: 0.222 g (0.29 mmol, 87 %).

^{1}H NMR (300 MHz, THF-d^8): δ 7.52 (t, J = 7.6 Hz, 1H, aryl), 7.35 (d, J = 7.6 Hz, 2H, aryl), 6.01–5.90 (m, phenazine CH^3 and CH^2), 5.77 (dd, J = 7.3 and 1.4 Hz, 2H, phenazine CH^4), 5.58 (dd, J = 7.3 and 1.4 Hz, 2H, phenazine CH^1), 4.09 (d, J = 12.3 Hz, 2H, CH_2), 2.88 (sept, J = 6.6 Hz, 2H, C*H*Me$_2$), 2.60 (s, 3H, NMe), 2.44–1.79 (m, 14H, adamantyl CH and CH_2), 1.38 (s, 6H, C(C$\underline{H_3}$)), 1.36 (d, J = 6.6 Hz, 6H, CH*Me*$_2$), 1.30 (d, J = 6.6 Hz, 6H, CH*Me*$_2$). ^{13}C NMR (75 MHz, THF-d^8) δ 242.9 (C carbene), 147.5 (*ipso*-CN phenazine), 146.0 (*o*-C), 140.0 (*ipso*-CNMe phenazine), 136.6 (*ipso*-C), 130.0 (*p*-CH), 125.8 (*m*-CH), 120.6 (phenazine CH), 117.9 (phenazine CH), 114.9 (phenazine CH^1), 109.5 (phenazine CH^4), 77.2 (C_q), 64.6 (C_q), 49.0 (CH_2), 39.8 (CH_2), 37.8 (CH), 35.4 (CH_2), 35.1 (CH_2), 31.3 (NMe), 29.7, 29.1, 28.7, 28.3, 26.3, 23.4 (CH_3). Anal. Calcd. for $C_{40}H_{50}N_3Au$ (769.81): C, 62.41; H, 6.55; N, 5.46. Found: C, 62.61; H, 6.70; N, 5.56.

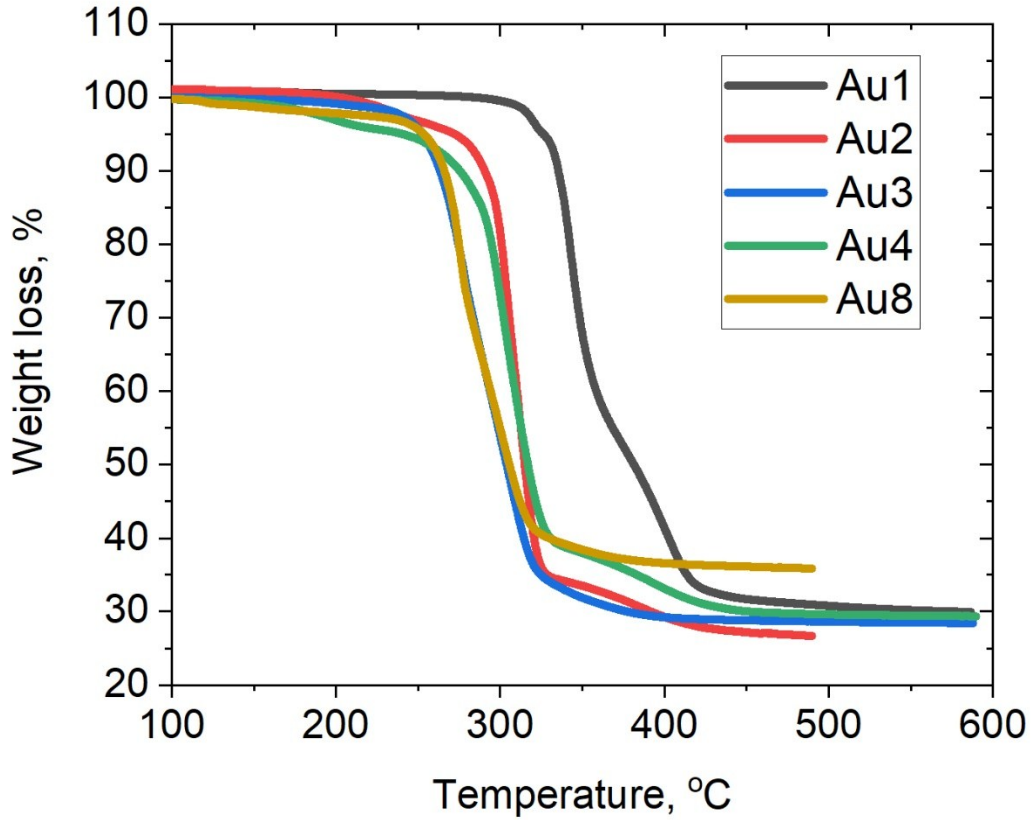

Figure S1. TGA curves for **Au1**, **Au2**, **Au3, Au4** and **Au8** complexes.

X-Ray Crystallography

Crystals of gold and copper complexes suitable for X-ray diffraction study were obtained by layering a CH_2Cl_2 or toluene solutions with hexane. Single crystals were mounted in oil on glass fiber and fixed on the diffractometer in a cold nitrogen stream. For the final refinement, the contribution of severely disordered CH_2Cl_2 molecules in the crystals of **Au4** was removed from the diffraction data with PLATON/SQUEEZE. [4,5] Monoclinic form of complex **Au1** was obtained by layering CH_2Cl_2 solution with hexane and crystallizes with two independent molecules and one CH_2Cl_2 solvent molecule in the unit cell. The CH_2Cl_2 molecule was disordered over two half-populated positions. Orthorhombic form of complex **Au1** was obtained by layering a toluene solution with hexane. Data were collected using an Oxford Diffraction Xcalibur-3/Sapphire3-CCD diffractometer with graphite monochromated Mo K_α radiation (λ = 0.71073 Å) at 140 K. Data were processed using the CrystAlisPro-CCD and – RED software.[6] The structure was solved by direct methods and refined by the full-matrix least-squares against F^2 in an anisotropic (for non-hydrogen atoms) approximation. All hydrogen atom positions were refined in isotropic approximation in "riding" model with the U_{iso}(H) parameters equal to 1.2 $U_{eq}(C_i)$, for methyl groups equal to 1.5 $U_{eq}(C_{ii})$, where $U(C_i)$

and $U(C_{ii})$ are respectively the equivalent thermal parameters of the carbon atoms to which the corresponding H atoms are bonded. All calculations were performed using the SHELX software.[7]

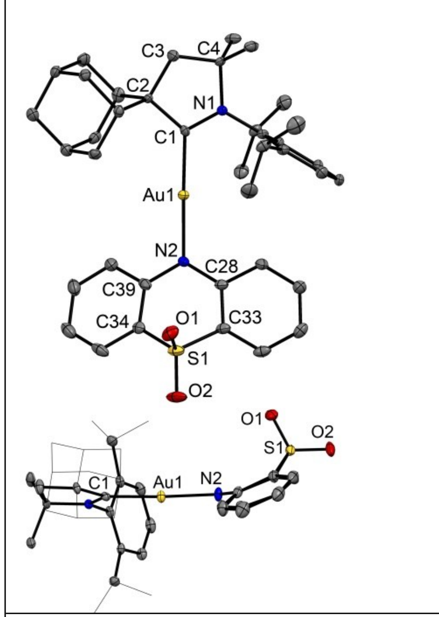

Au1·0.5CH$_2$Cl$_2$

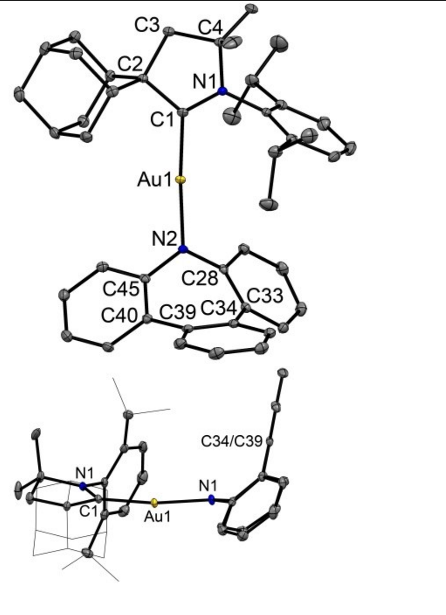

Au2

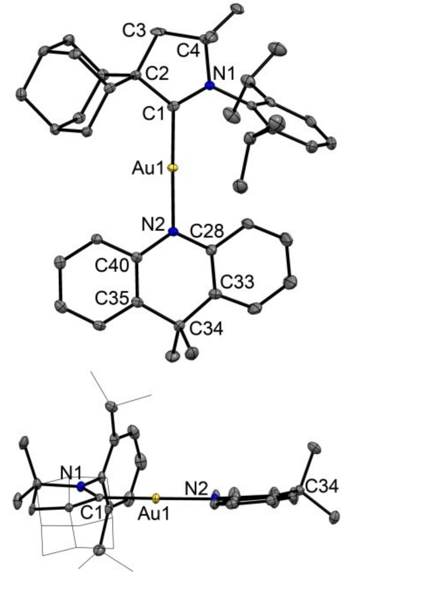

Au4

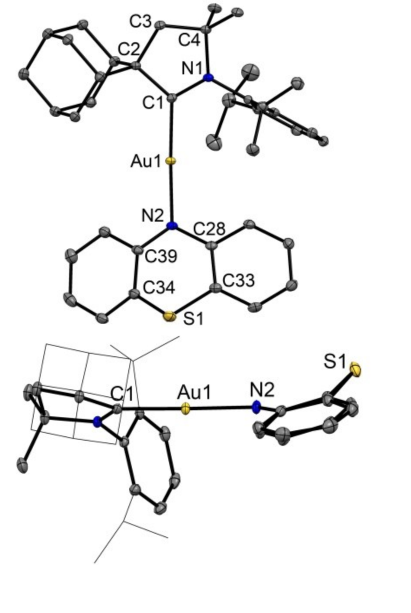

Au5

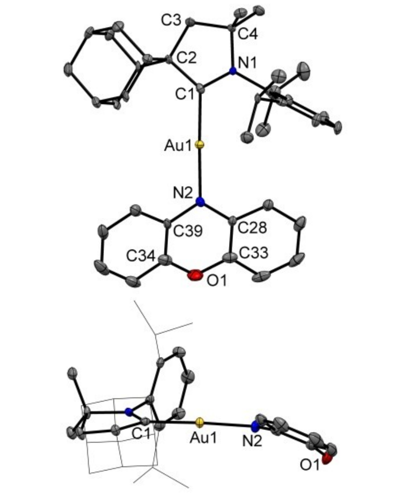

Au6

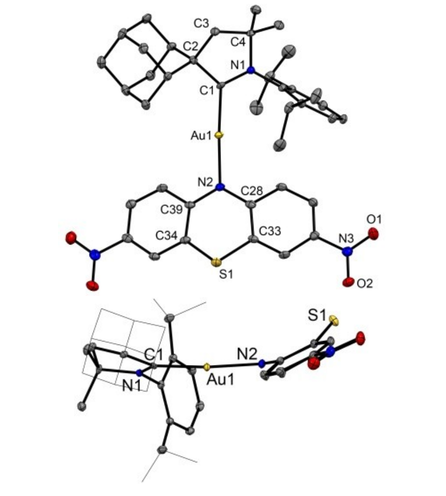

Au7

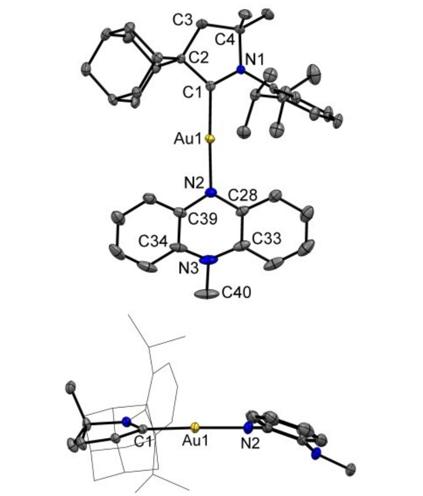

Au8

Figure S2. Crystal structures of carbene metal amides in top and side view, showing the conformations adopted by the amide ligands. The diagram of **Au1** depicts the monoclinic form. Ellipsoids are shown at the 50% level. Hydrogen atoms are omitted for clarity. For structural parameters, see Table 1 main text.

Complex **Au1** (Monoclinic) was obtained by layering CH_2Cl_2 solution with hexane: CCDC number 1912300, $C_{79}H_{96}Au_2Cl_2N_4O_4S_2$, Monoclinic, space group *P2₁/c*, a = 21.9650(5) Å, b = 16.1816(3) Å, c = 20.6249(7) Å, β = 99.929(3)°, V = 7220.9(3) Å^3, Z = 4, d_{calc} = 1.559 g cm^{-3}, μ = 4.243 mm^{-1}, colourless/block, crystal size 0.29 × 0.25 × 0.13 mm, F(000) = 3416.0, T_{min}/T_{max} 0.80026/1.00000, 76003 reflections measured (6.184° ≤ 2Θ ≤ 55.994°), 17391 unique (R_{int} = 0.0371, R_{sigma} = 0.0383) which were used in all calculations. The final R_1 was 0.0545 (I > 2σ(I)) and wR_2 was 0.1730 (all data), GOF = 1.325, $\Delta\rho_{min}/\Delta\rho_{max}$ = 3.06/–2.21.

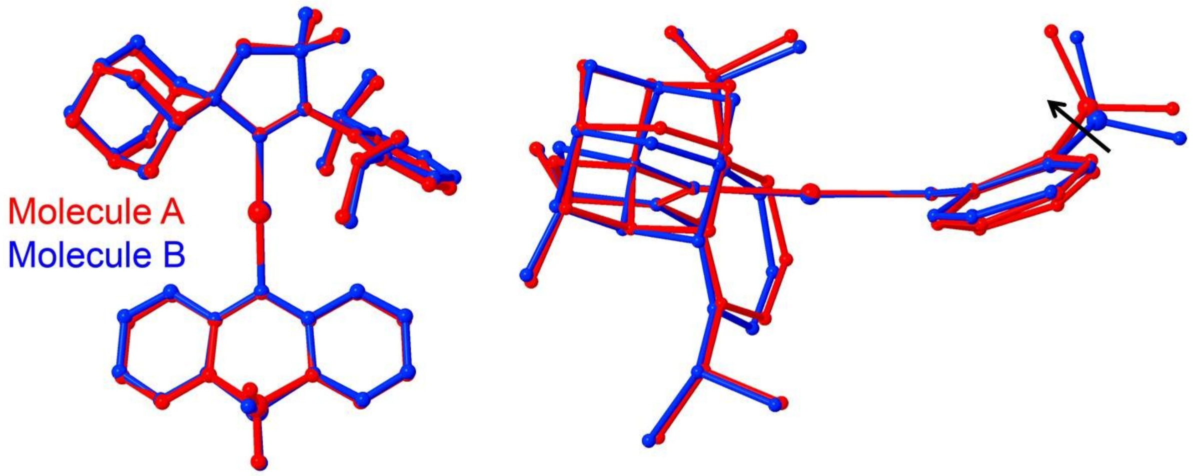

Figure S3. Overlap of independent molecules A and B for monoclinic form of **Au1**.

Complex **Au2**: CCDC number 1912299, $C_{45}H_{51}AuN_2$, Monoclinic, space group *P2₁/c*, a = 12.6669(4) Å, b = 17.4884(5) Å, c = 17.3107(5) Å, β = 108.451(3)°, V = 3637.6(2) Å^3, Z = 4, d_{calc} = 1.492 g cm^{-3}, μ = 4.078 mm^{-1}, yellow/block, crystal size 0.29 × 0.23 × 0.11 mm, F(000) = 1656.0, T_{min}/T_{max} 0.61278/1.00000, 38084 reflections measured (6.686° ≤ 2Θ ≤ 55.996°), 8778 unique (R_{int} = 0.0422, R_{sigma} = 0.0340) which were used in all calculations. The final R_1 was 0.0229 (I > 2σ(I)) and wR_2 was 0.0567 (all data), GOF = 1.027, $\Delta\rho_{min}/\Delta\rho_{max}$ = 0.98/–0.72.

Complex **Au4**: CCDC number 1911240, $C_{42}H_{53}AuN_2$, Orthorhombic, space group *Pbcn*, a = 34.2921(8) Å, b = 13.9716(3) Å, c = 17.0268(4) Å, V = 8157.8(3) Å^3, Z = 8, d_{calc} = 1.275 g cm^{-3}, μ = 3.634 mm^{-1}, orange/block, crystal size 0.22 × 0.19 × 0.06 mm, F(000) = 3184.0, T_{min}/T_{max} 0.60996/1.00000, 72878 reflections measured (6.086° ≤ 2Θ ≤ 56.0°), 9844 unique (R_{int} = 0.0700, R_{sigma} = 0.0419) which were used in all calculations. The final R_1 was 0.0353 (I > 2σ(I)) and wR_2 was 0.0874 (all data), GOF = 1.090, $\Delta\rho_{min}/\Delta\rho_{max}$ = 1.66/–0.83.

Complex **Au5**: CCDC number 1911241, $C_{39}H_{47}AuN_2S$, Monoclinic, space group *P2*$_1$*/c*, a = 12.6173(10) Å, b = 13.9286(6) Å, c = 19.6070(9) Å, β = 108.245(7)°, V = 3272.5(4) Å^3, Z = 4, d_{calc} = 1.569 g cm^{-3}, μ = 4.590 mm^{-1}, yellow/block, crystal size 0.35 × 0.22 × 0.16 mm, F(000) = 1560.0, T_{min}/T_{max} 0.80026/1.00000, 32402 reflections measured (6.246° ≤ 2Θ ≤ 55.998°), 7887 unique (R_{int} = 0.0226, R_{sigma} = 0.0196) which were used in all calculations. The final R_1 was 0.0182 (I > 2σ(I)) and wR_2 was 0.0489 (all data), *GOF* = 1.046, $\Delta\rho_{min}/\Delta\rho_{max}$ = 1.09/–0.43.

Complex **Au6**: CCDC number 1911239, $C_{40}H_{49}AuCl_2N_2O$, Orthorhombic, space group *Pbca*, a = 10.0502(2) Å, b = 17.6546(3) Å, c = 39.3833(6) Å, V = 6987.9(2) Å^3, Z = 8, d_{calc} = 1.600g cm^{-3}, μ = 4.398 mm^{-1}, red/prism, crystal size 0.33 × 0.19 × 0.16 mm, F(000) = 3392, T_{min}/T_{max} 0.51653/1.00000, 64151 reflections measured (6.23° ≤ 2Θ ≤ 52.0°), 6853 unique (R_{int} = 0.0528, R_{sigma} = 0.0275) which were used in all calculations. The final R_1 was 0.0652 (I > 2σ(I)) and wR_2 was 0.1048 (all data), *GOF* = 1.461, $\Delta\rho_{min}/\Delta\rho_{max}$ = 2.31/–4.04.

Complex **Au7**: CCDC number 1911237, $C_{39}H_{45}AuN_4O_4S$, Triclinic, space group *P-1*, a = 10.7063(2) Å, b = 13.5187(3) Å, c = 13.5554(2) Å, α = 71.713(2)°, β = 76.289(2)°, γ = 70.466(2)°, V = 1736.40(6) Å^3, Z = 2, d_{calc} = 1.650 g cm^{-3}, μ = 4.344 mm^{-1}, red/plate, crystal size 0.39 × 0.25 × 0.08 mm, F(000) = 868.0, T_{min}/T_{max} 0.64935/1.00000, 16604 reflections measured (5.934° ≤ 2Θ ≤ 56°), 8360 unique (R_{int} = 0.0303, R_{sigma} = 0.0466) which were used in all calculations. The final R_1 was 0.0270 (I > 2σ(I)) and wR_2 was 0.0597 (all data), *GOF* = 0.975, $\Delta\rho_{min}/\Delta\rho_{max}$ = 1.83/– 1.94.

Complex **Au8**: CCDC number 1911238, $C_{41}H_{52}AuCl_2N_3$, Orthorhombic, space group *Pbca*, a = 10.4512(1) Å, b = 18.0543(2) Å, c = 38.7653(5) Å, V = 7314.59(14) Å^3, Z = 8, d_{calc} = 1.552 g cm^{-3}, μ = 4.202 mm^{-1}, red/plate, crystal size 0.32 × 0.17 × 0.08 mm, F(000) = 3456, T_{min}/T_{max} 0.82172/1.00000, 122432 reflections measured (6.056° ≤ 2Θ ≤ 55.998°), 8809 unique (R_{int} = 0.0324, R_{sigma} = 0.0131) which were used in all calculations. The final R_1 was 0.0266 (I > 2σ(I)) and wR_2 was 0.0851 (all data), *GOF* = 1.065, $\Delta\rho_{min}/\Delta\rho_{max}$ = 1.72/– 1.83.

Electrochemistry.

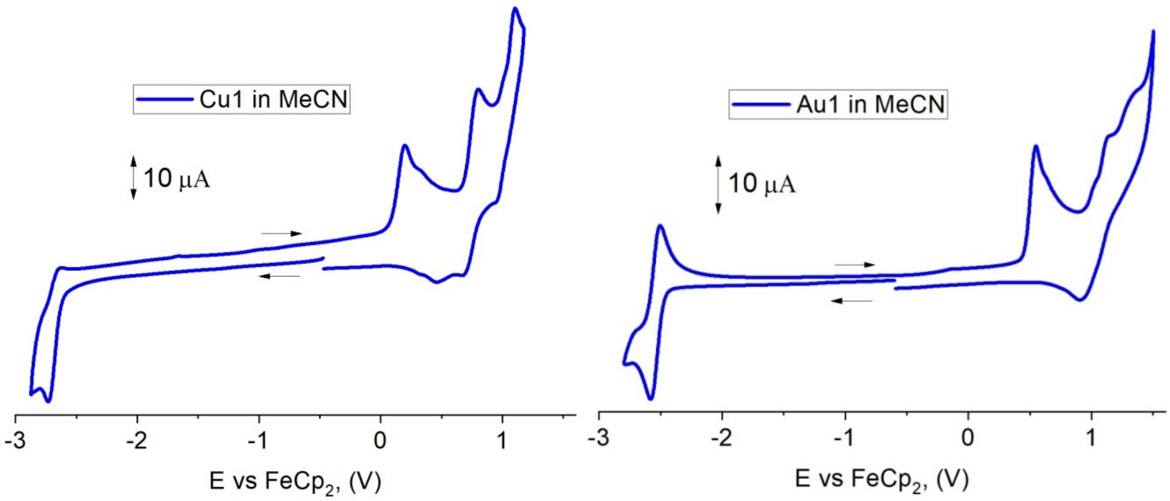

Figure S4. Full range cyclic voltammograms of complexes **Cu1** (left) and **Au1** (right). Recorded using a glassy carbon electrode in MeCN solution (1.4 mM) with $[n\text{-}Bu_4N]PF_6$ as supporting electrolyte (0.13 M), scan rate 0.1 V s^{-1}.

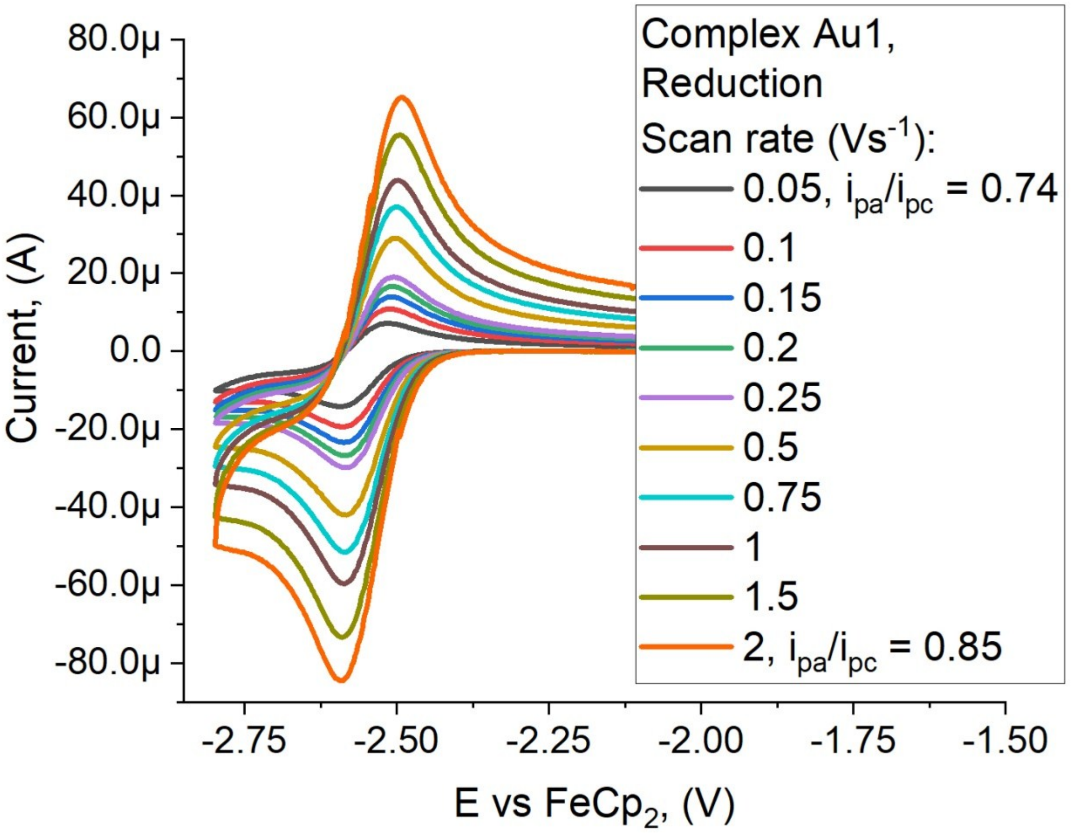

Figure S5. Varied scan rate behaviour for the reduction process of complex **Au1**. Recorded using a glassy carbon electrode in MeCN solution (1.4 mM) with $[n\text{-}Bu_4N]PF_6$ as supporting electrolyte (0.13 M) and scan rates from 0.05 to 2 V s^{-1}.

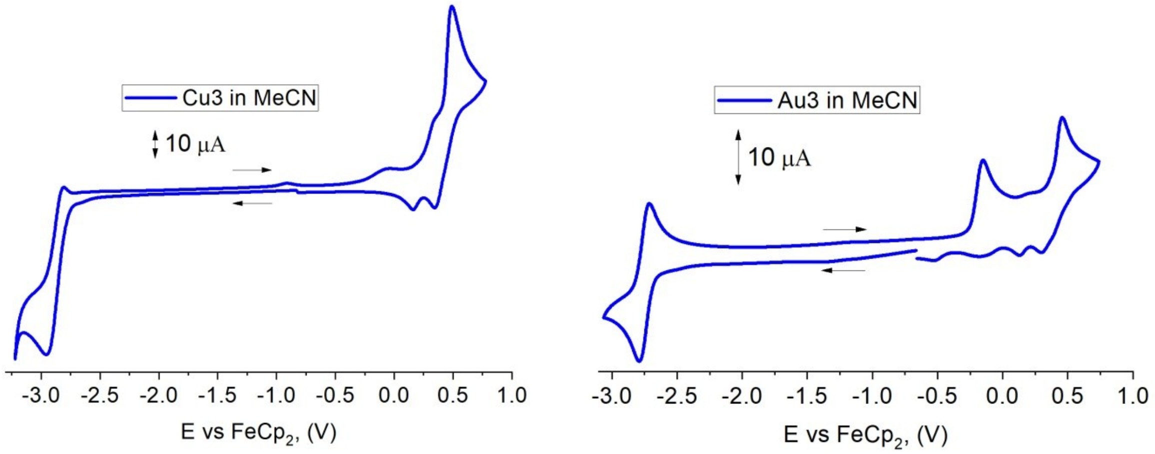

Figure S6. Full range cyclic voltammograms of complexes **Cu3** (left) and **Au3** (right). Recorded using a glassy carbon electrode in MeCN solution (1.4 mM) with [n-Bu_4N]PF_6 as supporting electrolyte (0.13 M), scan rate 0.1 V s^{-1}.

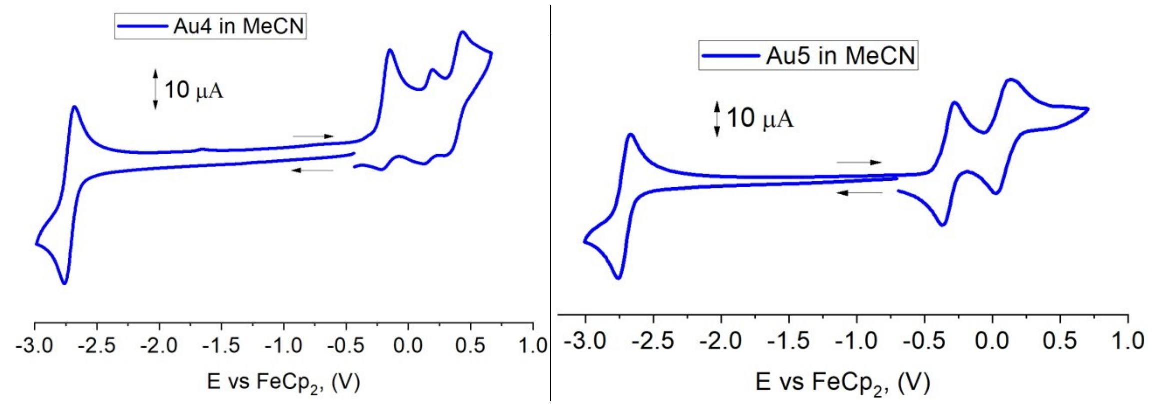

Figure S7. Full range cyclic voltammograms of complexes **Au4** (left) and **Au5** (right). Recorded using a glassy carbon electrode in MeCN solution (1.4 mM) with [n-Bu_4N]PF_6 as supporting electrolyte (0.13 M), scan rate 0.1 V s^{-1}.

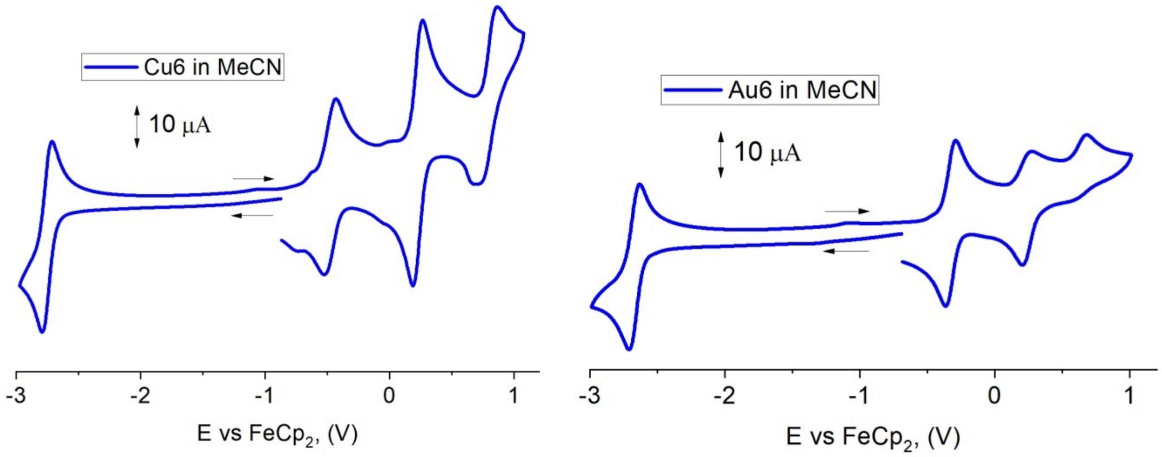

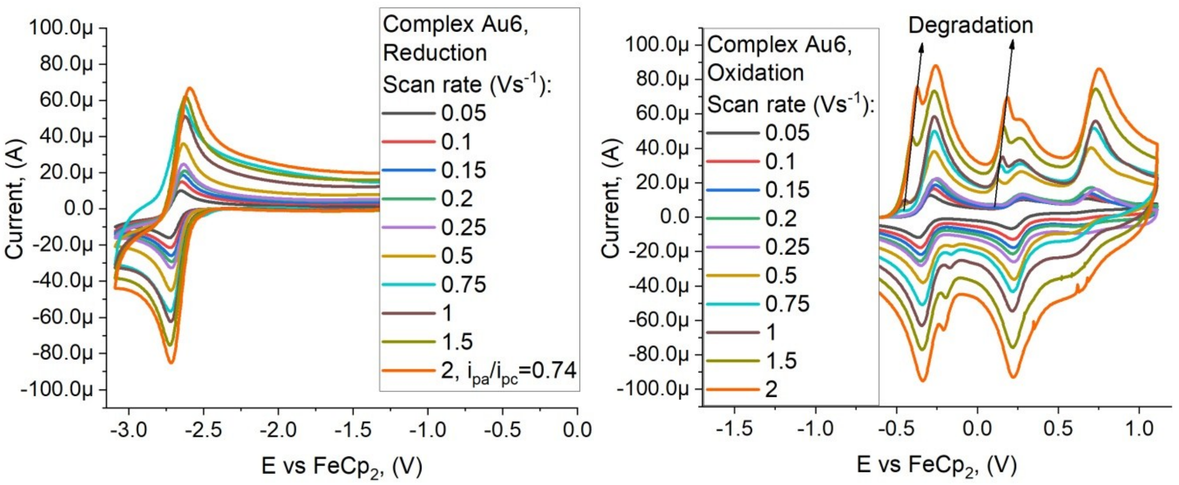

Figure S8. Top: full range cyclic voltammograms of complexes **Cu6** (left) and **Au6** (right). Recorded using a glassy carbon electrode in MeCN solution (1.4 mM) with [n-Bu_4N]PF_6 as supporting electrolyte (0.13 M), scan rate 0.1 V s^{-1}. Bottom: varied scan rate behaviour for reduction (left) and oxidation (right) processes for complex **Au6**, indicating better stability on reduction rather than oxidation scans.

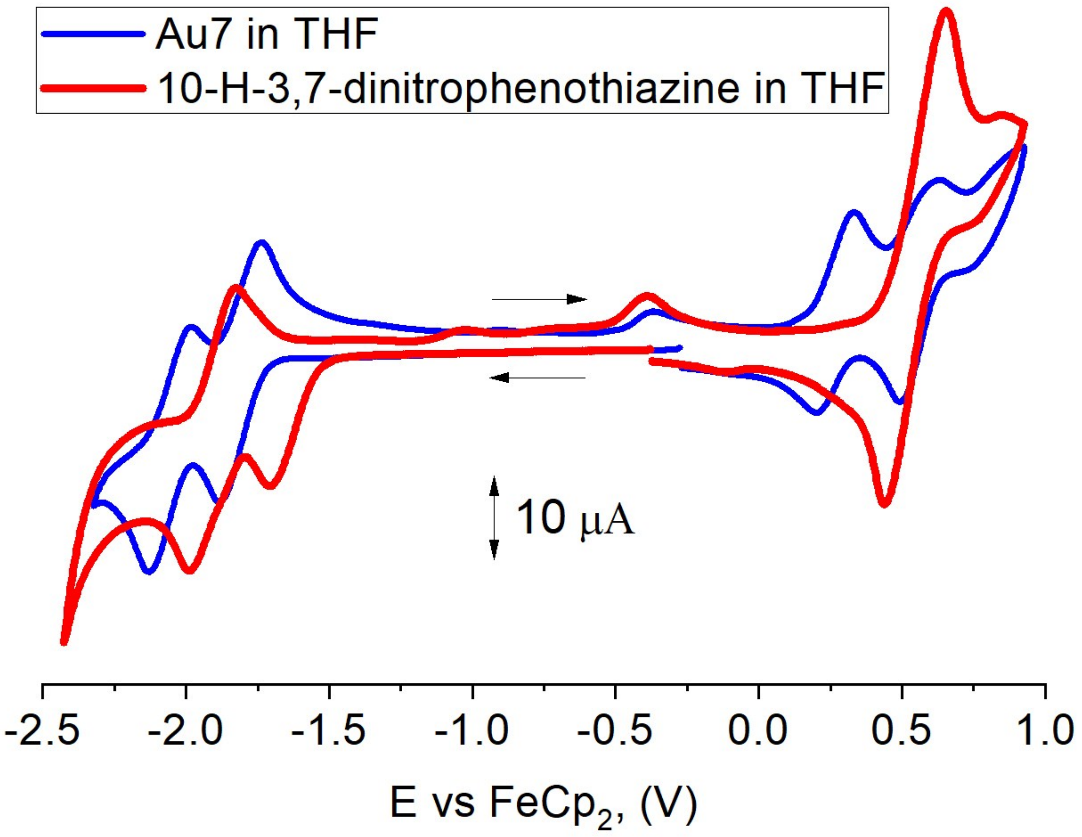

Figure S9. Full range cyclic voltammogram for complex **Au7** (blue line) and precursor 10-H-3,7-dinitrophenothiazine (red line). Recorded using a glassy carbon electrode in THF solution

(1.4 mM) with [n-Bu_4N]PF_6 as supporting electrolyte (0.13 M), scan rate 0.1 V s^{-1}. Note, THF solution was used due to poor solubility of complex **Au7** in MeCN.

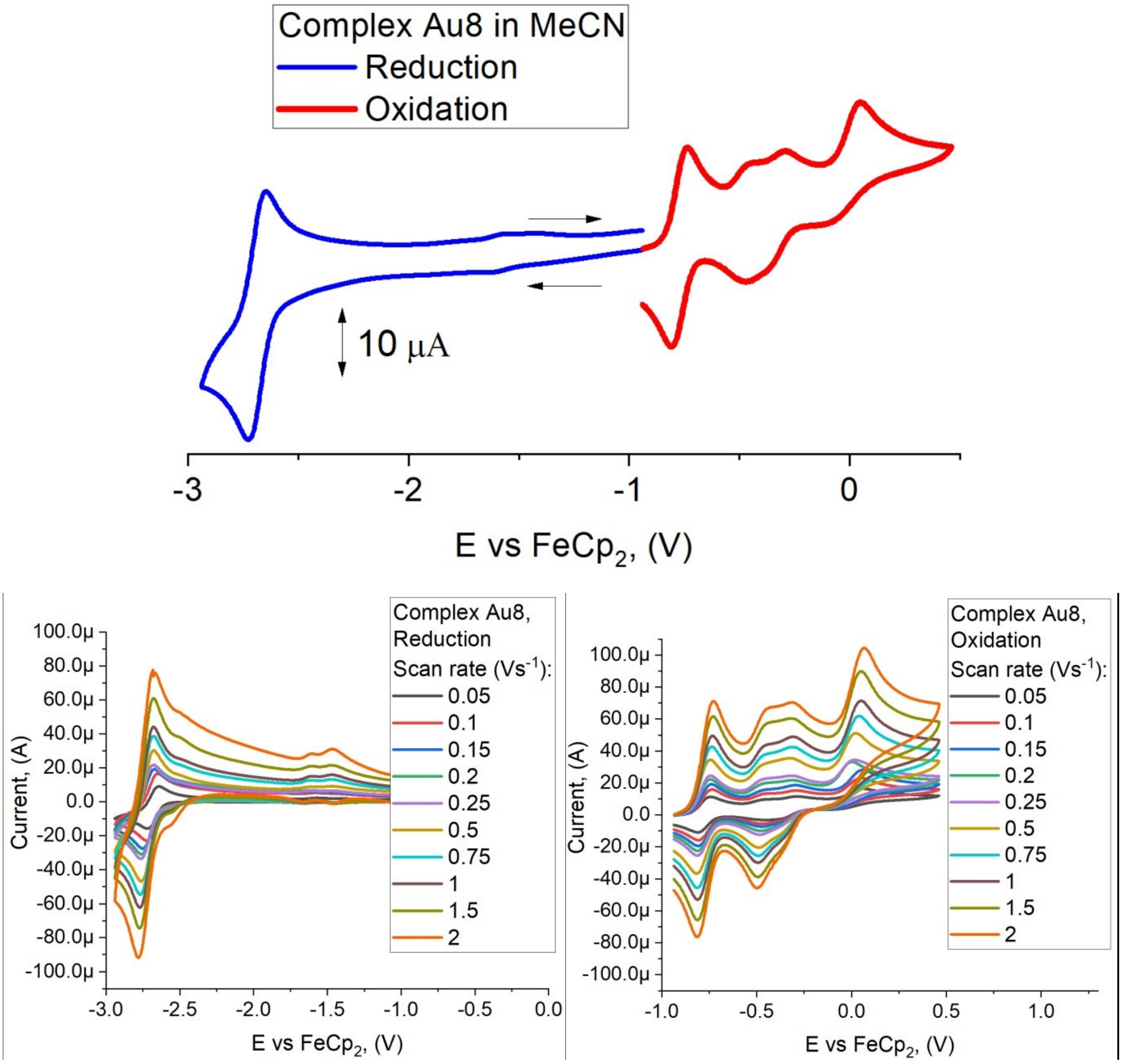

Figure S10. Top: full range cyclic voltammogram for complex **Au8**. Recorded using a glassy carbon electrode in MeCN solution (1.4 mM) with [n-Bu_4N]PF_6 as supporting electrolyte (0.13 M), scan rate 0.1 V s^{-1}. Bottom: varied scan rate behaviour for reduction (left) and oxidation (right), indicating better stability on oxidation rather than reduction scans.

Table S1. Formal electrode potentials (peak position E_p for irreversible and $E_{1/2}$ for quasi-reversible processes (*), *V*, *vs*. $FeCp_2$), onset potentials (*E, V*, *vs*. $FeCp_2$), peak-to-peak separation in parentheses for quasi-reversible processes (ΔE_p in mV), E_{HOMO}/E_{LUMO} (*e*V) and band gap values (*ΔE*, *e*V) for the redox changes exhibited by copper and gold complexes.[a]

Complex	Reduction		E_{LUMO} eV	Oxidation				E_{HOMO} eV	ΔE eV
	E_{1st}	$E_{onset\ red}$		E_{1st}	$E_{onset\ ox}$	E_{2nd}	E_{3rd}		
**Cu1**	–2.73	–2.61	–2.78	+0.19	+0.06	+0.79	+1.09	–5.45	2.67
**Cu2**	–2.97	–2.84	–2.55	–0.32	–0.43	+0.48	+0.6	–4.96	2.41
**Cu3**	–2.88* (145)	–2.78	–2.61	–0.08	–0.28	+0.33	+0.41*	–5.11	2.50
**Cu6**	–2.75* (79)	–2.68	–2.71	–0.48* (84)	–0.59	+0.22* (80)	+0.78* (168)	–4.80	2.09
**Au1**	–2.54* (78)	–2.47	–2.92	+0.54	+0.46	+1.13	+1.34	–5.85	2.93
**Au2**	–2.88* (91)	–2.76	–2.63	–0.14	–0.22	+0.52	–	–5.17	2.54
**Au3**	–2.75* (77)	–2.67	–2.72	–0.15	–0.25	+0.45	–	–5.14	2.42
**Au4**	–2.72* (80)	–2.63	–2.76	–0.15	–0.25	+0.19	+0.43	–5.14	2.38
**Au5**	–2.71* (88)	–2.64	–2.75	–0.32* (84)	–0.42	+0.07* (106)	–	–5.07	2.32
**Au6**	–2.67* (84)	–2.60	–2.79	–0.33* (73)	–0.40	+0.24* (54)	+0.67	–4.99	2.20
**Au7**[b]	–1.81*(149) E_{2nd}= –2.05*	–1.73	–3.66	+0.26* (128)	+0.17	+0.56	–	–5.56	1.90

**Ligand_ 7**[c]	–1.71 E_{2nd}= –1.90*	–1.54	–3.85	+0.54* (214)	+0.44	–	–	–5.83	1.98
**Au8**	–2.70* (83)	–2.61	–2.78	–0.77* (66)	–0.83	–0.45	+0.01	–4.56	1.64

[a] In MeCN solution, recorded using a glassy carbon electrode, concentration 1.4 m*M*, supporting electrolyte [^{n}Bu$_4$N][PF$_6$] (0.13 *M*), measured at 0.1 V s$^{–1}$.

[b] Note, THF solution was used due to poor solubility of complex **Au7** in MeCN, recorded using a glassy carbon electrode, concentration 1.4 m*M*, supporting electrolyte [^{n}Bu$_4$N][PF$_6$] (0.13 *M*), measured at 0.1 V s^{-1}.

[c] Values for the organic amine ligand 10-H-3,7-dinitrophenothiazine in THF solution, recorded using a glassy carbon electrode, concentration 1.4 m*M*, supporting electrolyte [^{n}Bu$_4$N][PF$_6$] (0.13 *M*), measured at 0.1 V s^{-1}.

Photophysical Characterisation

UV-visible absorption spectra were recorded using a Perkin-Elmer Lambda 35 UV/vis spectrometer. Photoluminescence measurements were recorded on a Fluorolog Horiba Jobin Yvon spectrofluorimeter with a solids mount attachment where appropriate. Photoluminescence quantum yield, and additional photoluminescence spectra, were recorded using an Edinburgh Instruments FLS980 spectrometer. Quantum yields have been measured in air and under nitrogen for solid samples and under nitrogen for solutions.

Time resolved fluorescence data were collected on a time-correlated single photon counting (TCSPC) Fluorolog Horiba Jobin Yvon spectrofluorimeter using Horiba Jobin Yvon DataStation v2.4 software. A NanoLED of 370 nm was used as excitation source, with an instrument response function width of 2 ns. The collected data were analysed using a Horiba Jobin Yvon DAS6 v6.3 software.

UV-vis and Photoluminescence Spectra:

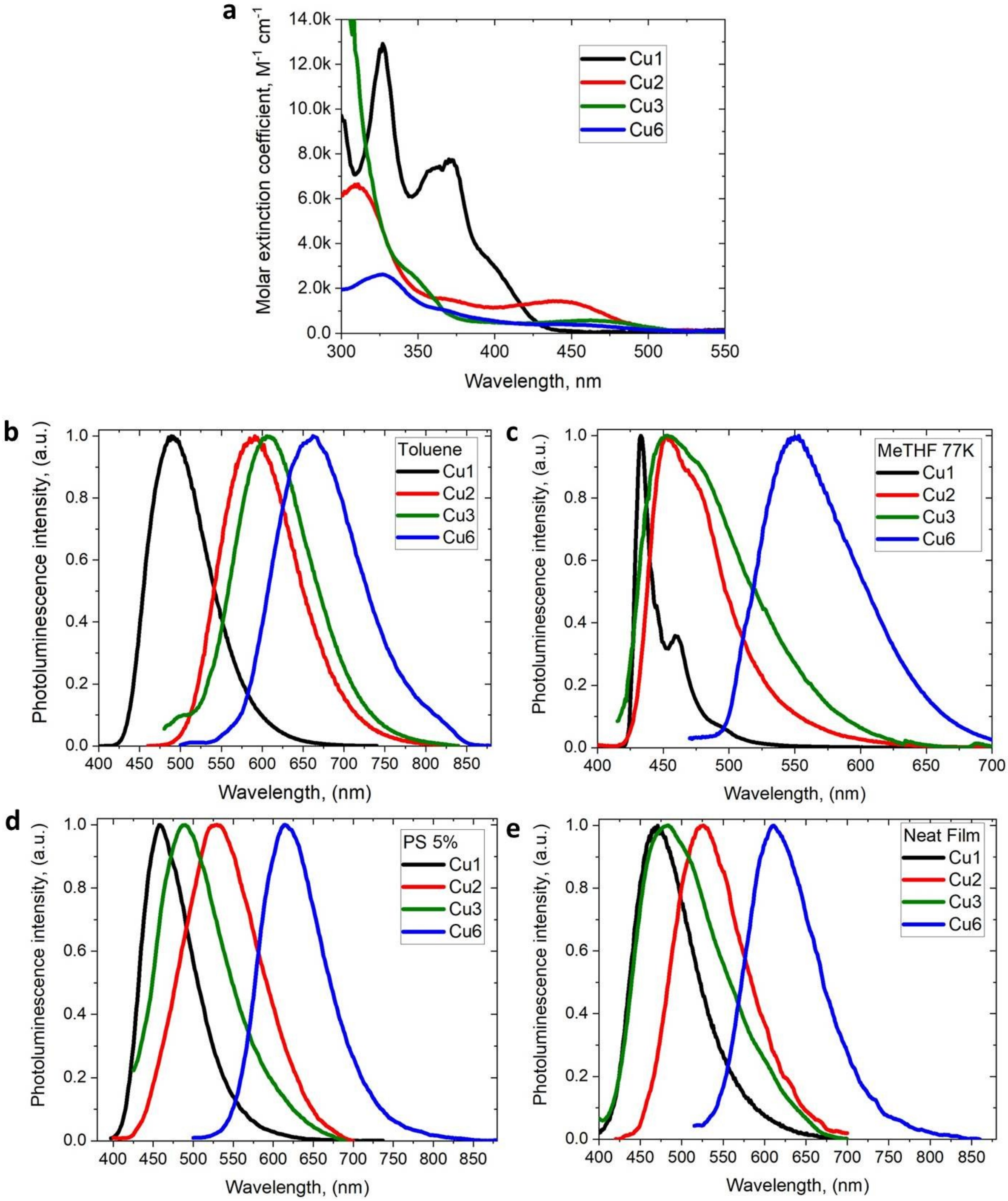

Figure S11. UV/vis spectra of copper **Cu1-Cu3** and **Cu6** (a). PL spectra of copper CMA complexes **Cu1**-**Cu3** and **Cu6** in toluene solution at 298 K (b), in MeTHF at 77 K (c), as a 5wt-% dopant in polystyrene (d), and as neat films under N_2 (e).

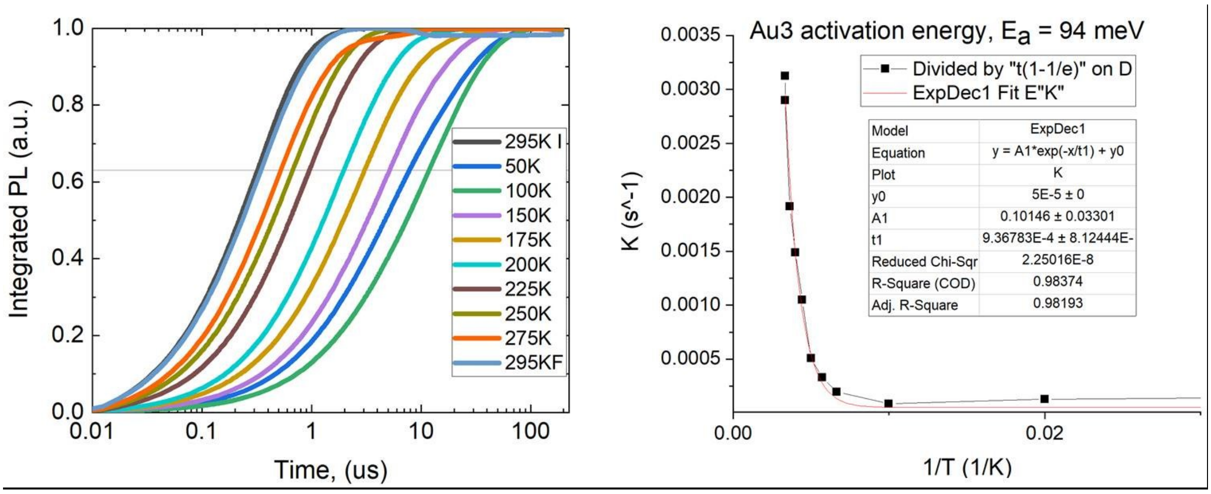

Figure S12. Left: temperature dependent integrated photoluminescence plots for complex **Au3** (295KI initial before cooling; 295KF final after warming). Right: Arrhenius plot to estimate thermal activation energy E_A of the characteristic decay rates k for **Au3**, assumed to be of the form $k = k_0 + k' e^{-\frac{E_A}{k_B T}}$

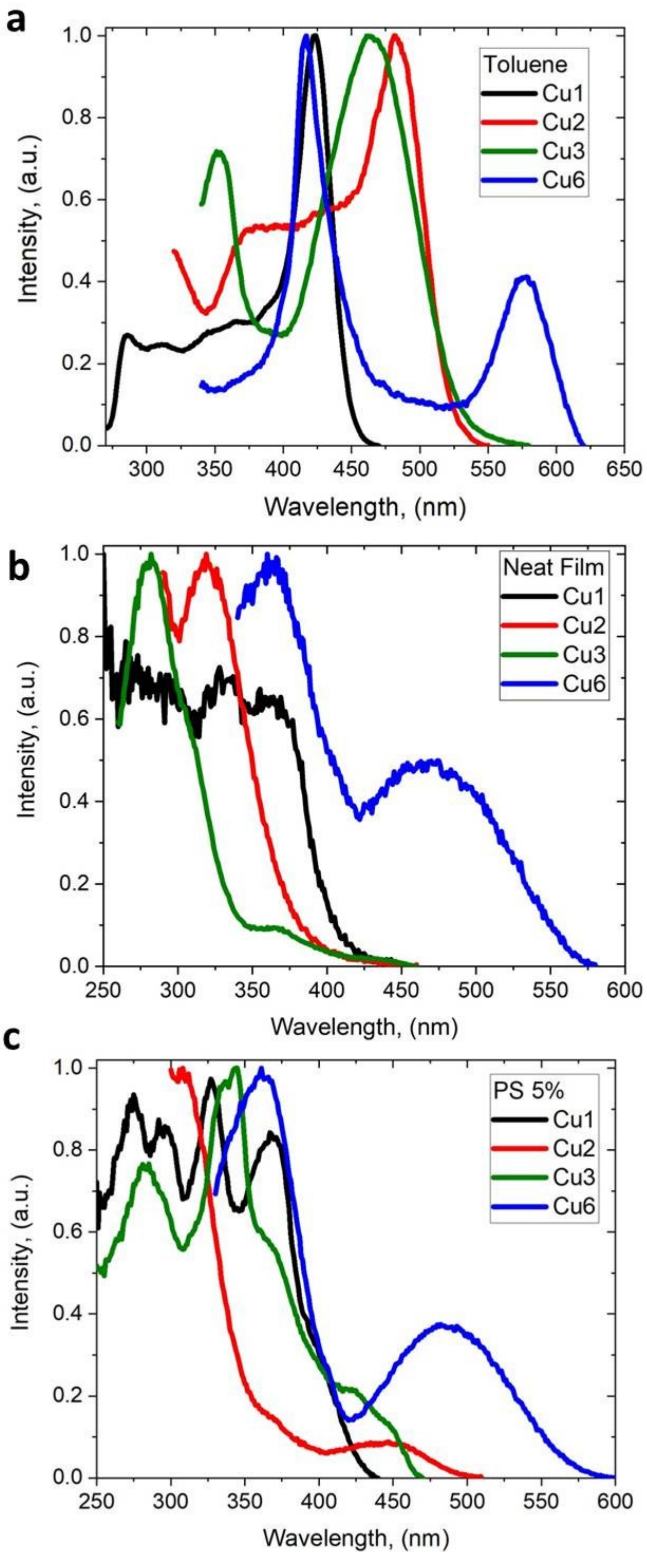

Figure S13. Excitation spectra in toluene (a), neat film (b) and PS 5% matrix (c) for copper complexes measured at the emission max.

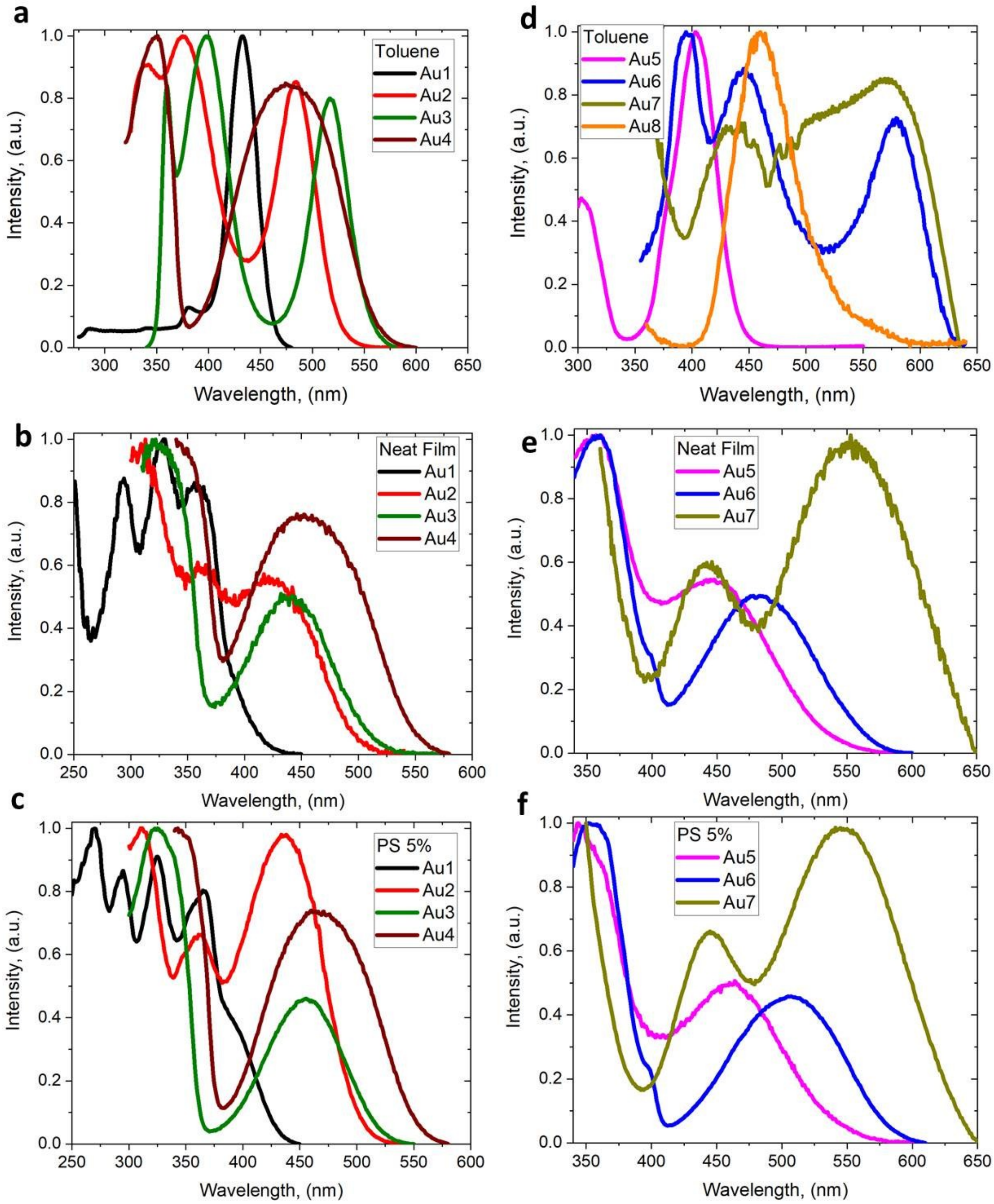

Figure S14. Excitation spectra in toluene (a, d), neat film (b, e) and PS 5% matrix (c, f) for gold complexes measured at the emission max.

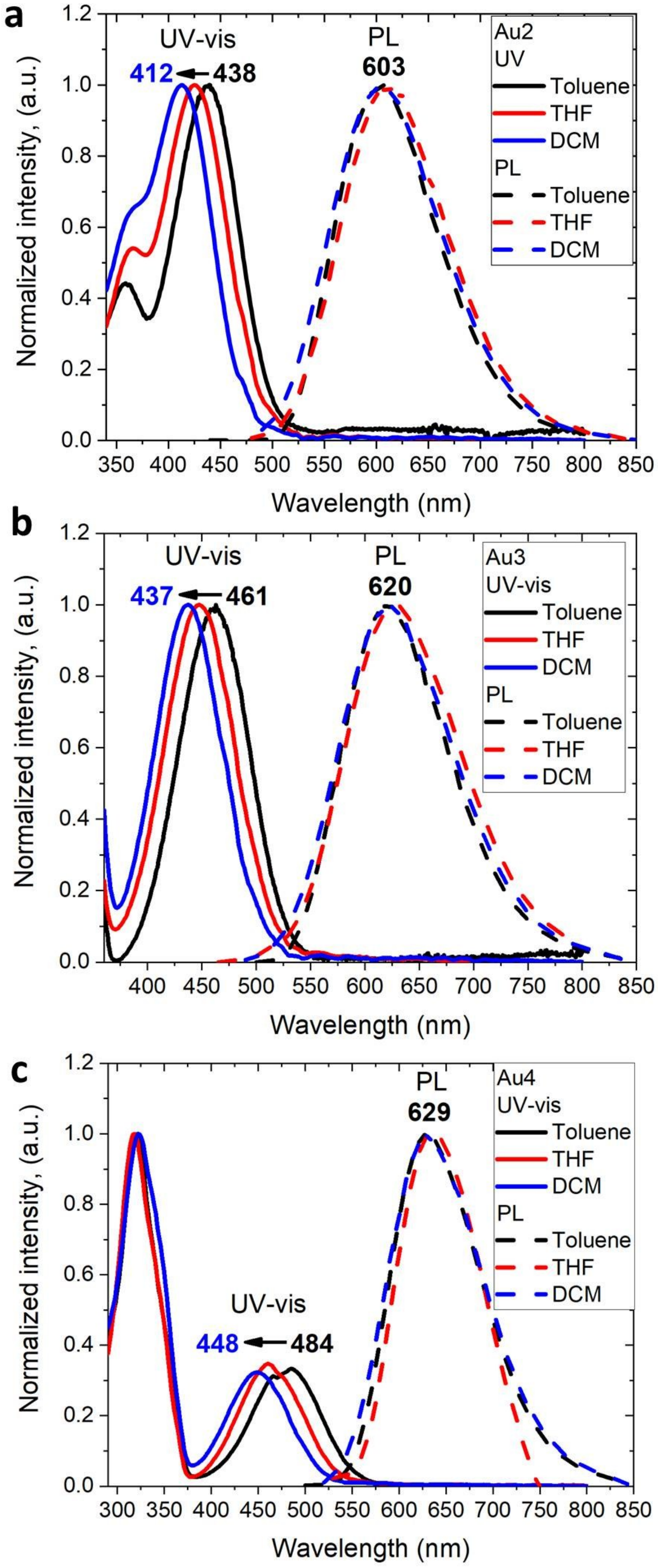

Figure S15. Normalized UV-vis and emission spectra in toluene, THF and CH_2Cl_2 for gold complex **Au2**, **Au3** and **Au4** showing negative solvatochromism.

Computational details.

The ground states of the complexes were studied by density functional theory (DFT) and the excited states by time-dependent DFT (TD-DFT) using the Tamm-Dancoff approximation.[8],[9] Calculations were carried by the global hybrid MN15 functional of the Minnesota series by Truhlar and coworkers, which has especially good performance for noncovalent interactions and excitation energies,[10] and as has been shown to perform well for closely related molecules.[11],[12] The MN15 method was combined with the def2-TZVP basis set by Ahlrichs and coworkers.[13],[14] Relativistic effective core potential of 60 electrons was used to describe the core electrons of Au.[15] All calculations were carried out by Gaussian 16.[16]

Table S2. Ground and excited state optimized structures for copper and gold complexes.

S0	S1	T1
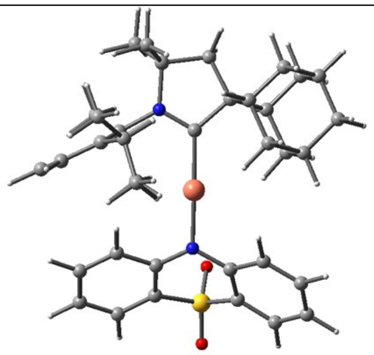 Cu1	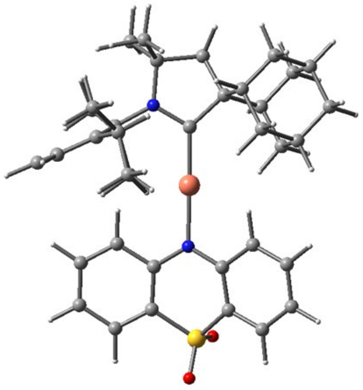	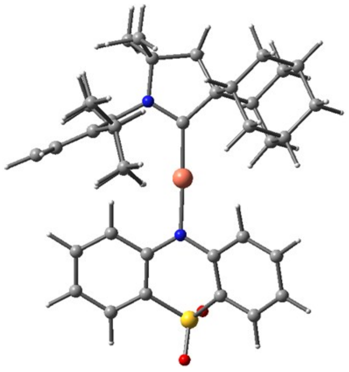
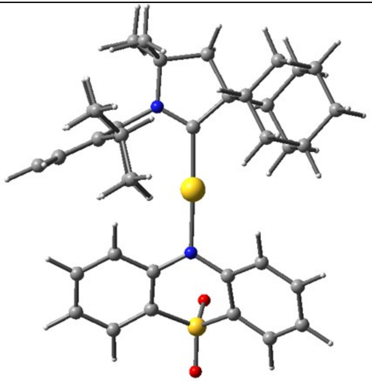 Au1	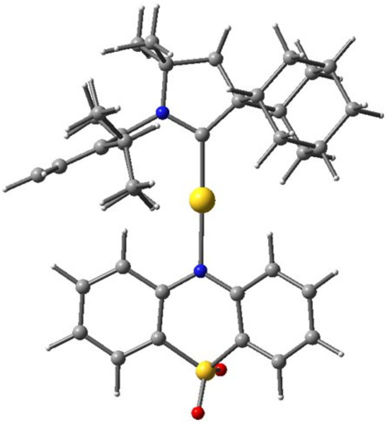	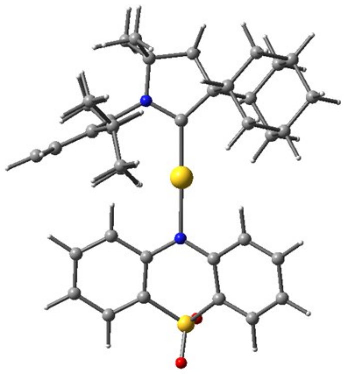

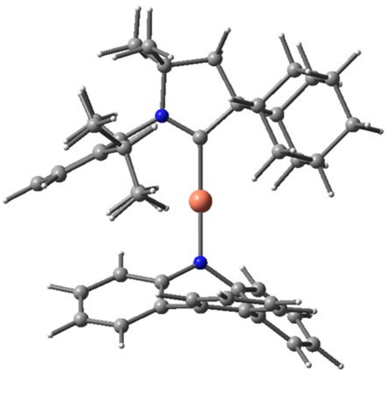 Cu2	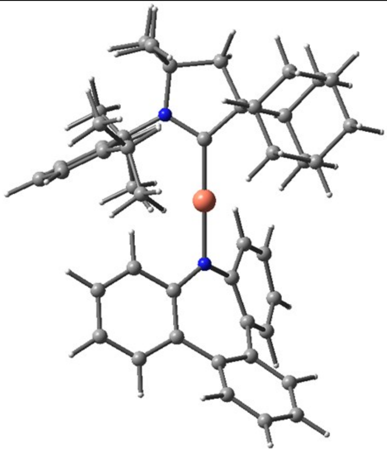	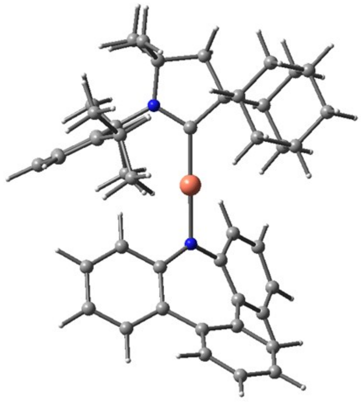
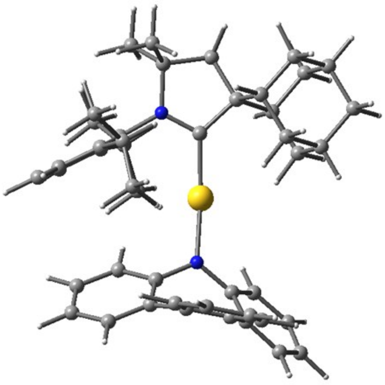 Au2	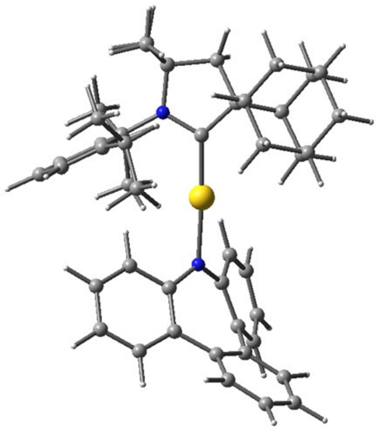	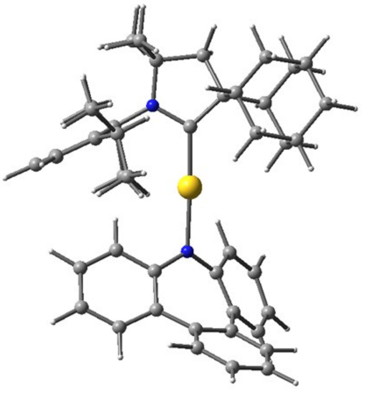
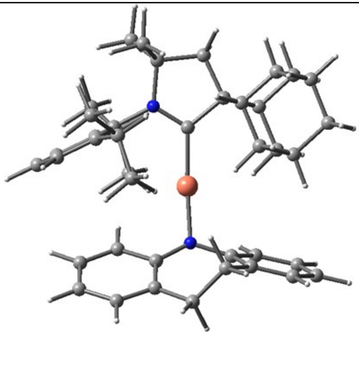 Cu3	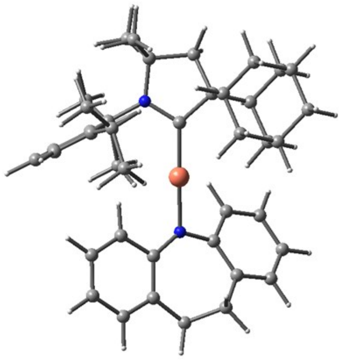	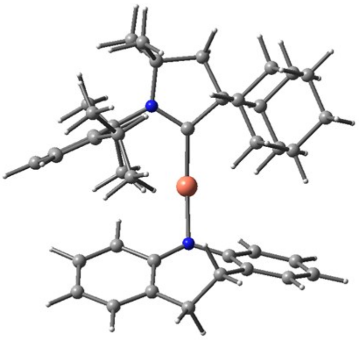
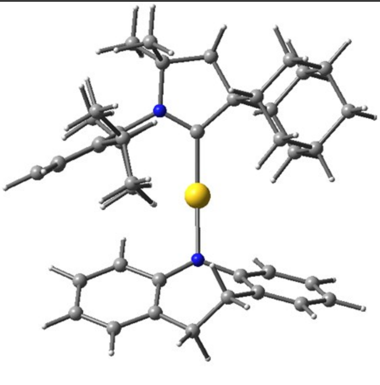 Au3	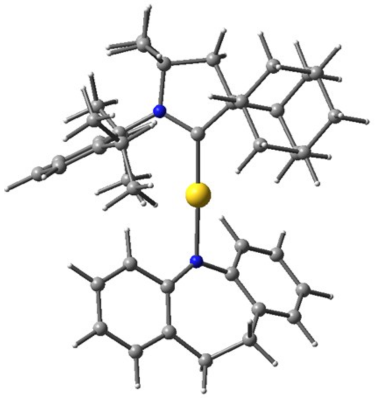	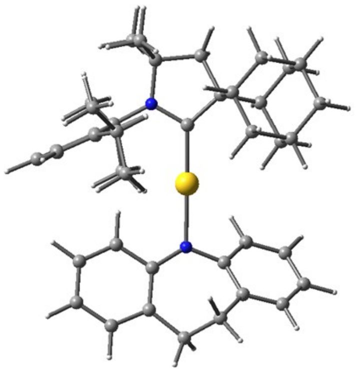

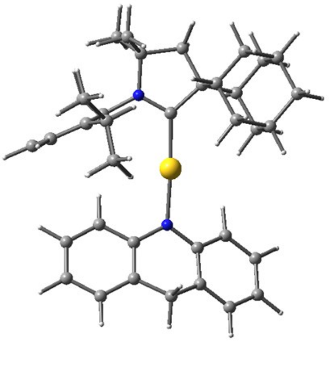<br>Au4	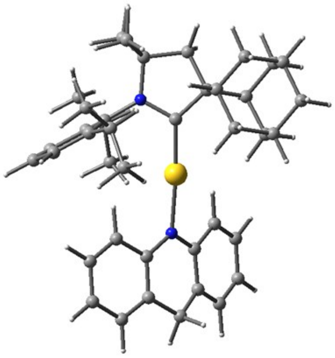	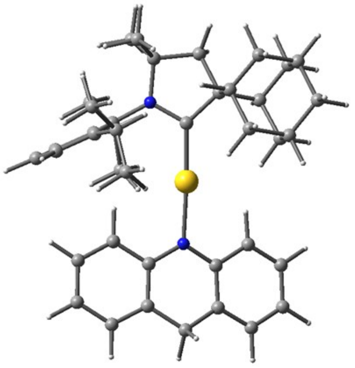
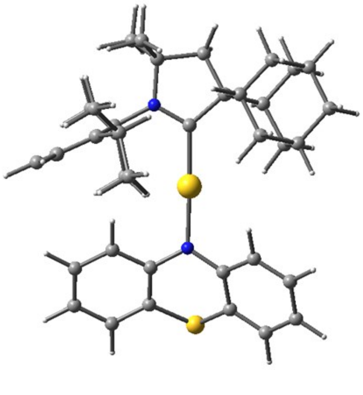<br>Au5	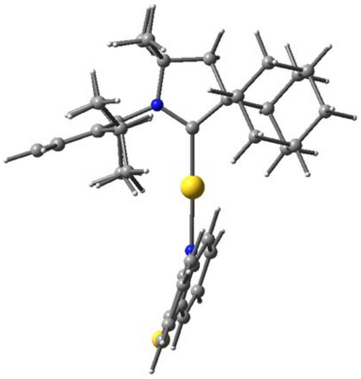	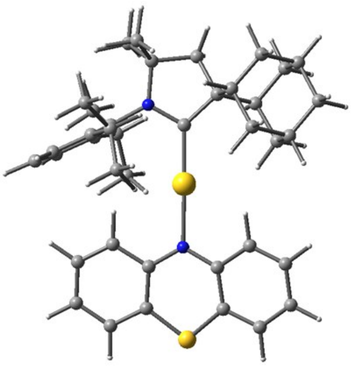
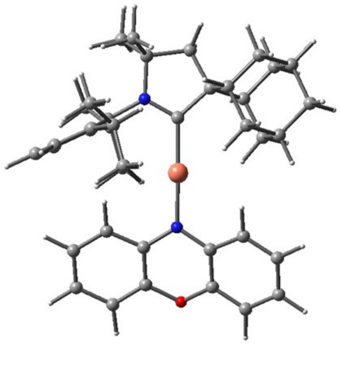<br>Cu6		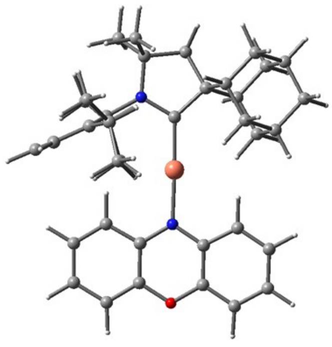
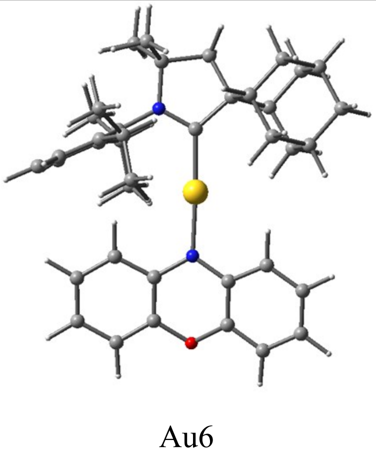<br>Au6	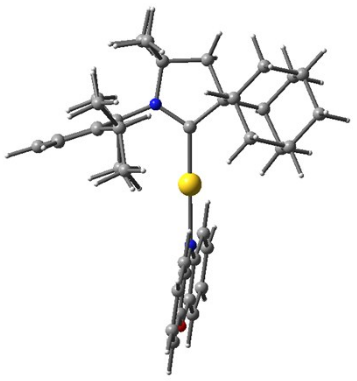	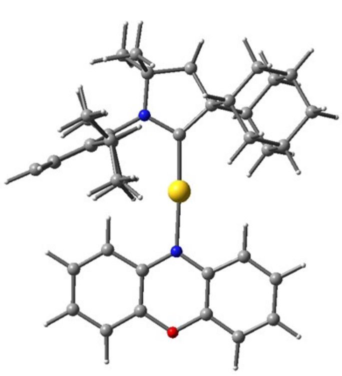

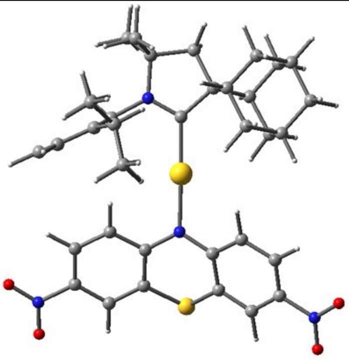<br>Au7	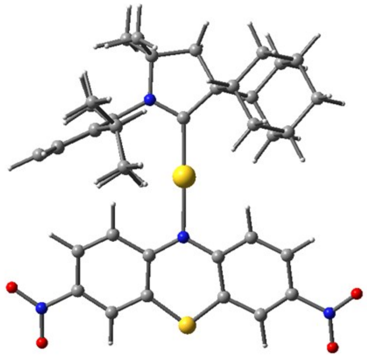	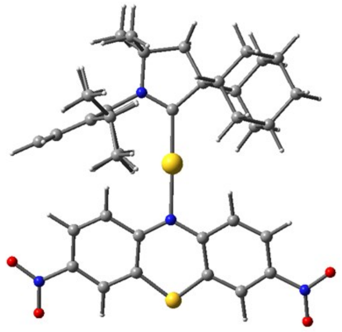
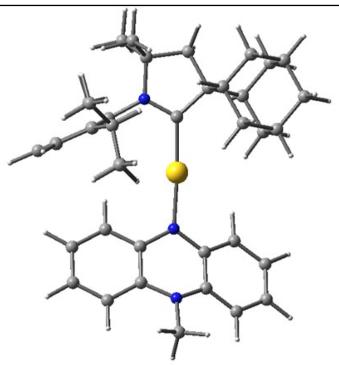<br>Au8	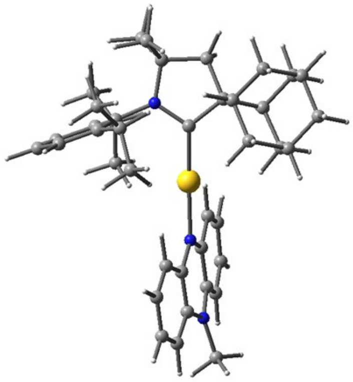	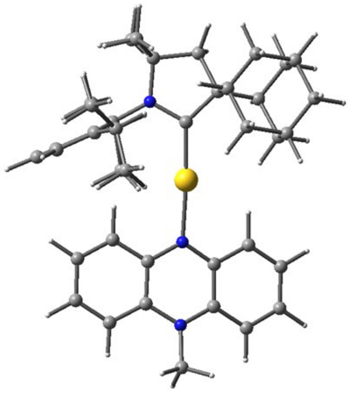

Table S3. HOMOs and LUMOs of CMA complexes

Optimized ground state structure	HOMO	LUMO
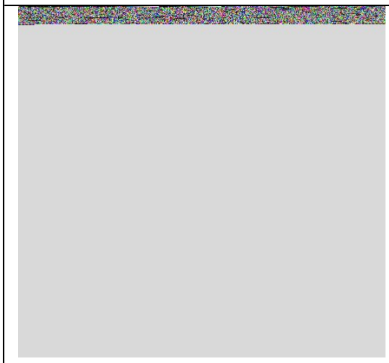 **Cu1**	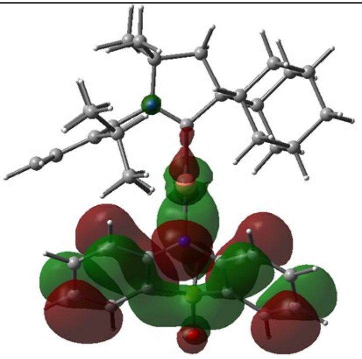 Cu: 4.4%	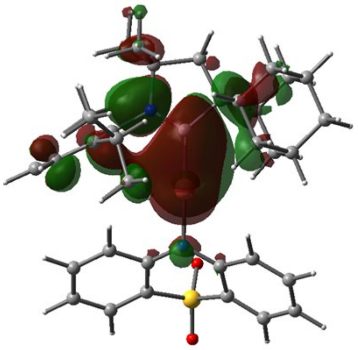 Cu: 7.5%
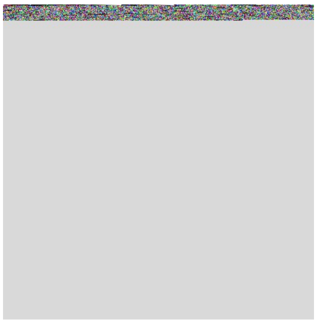 **Au1**	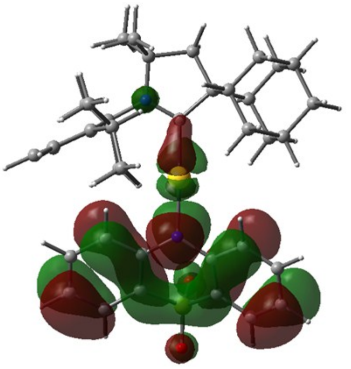 Au: 3.4%	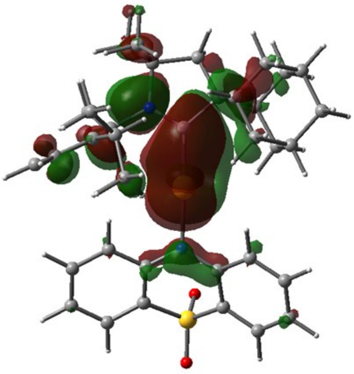 Au: 12.7%
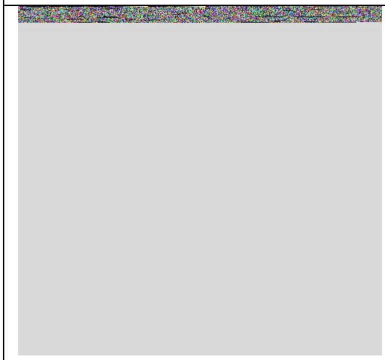 **Cu2**	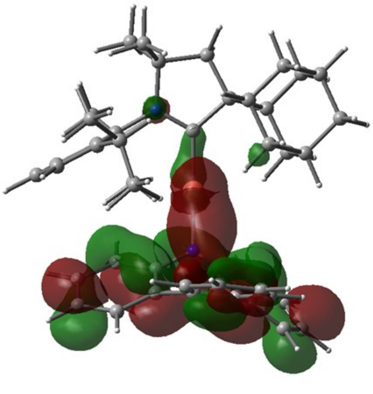 Cu: 6.0%	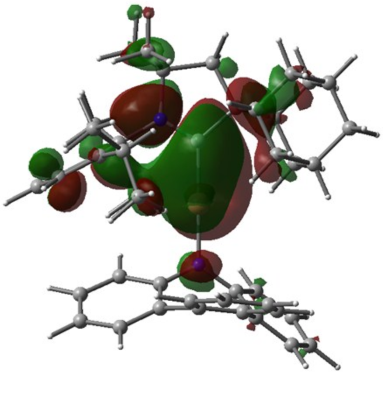 Cu: 9.2%

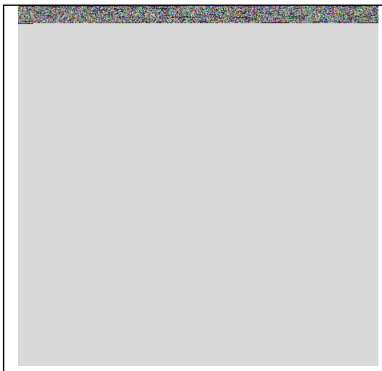 **Au2**	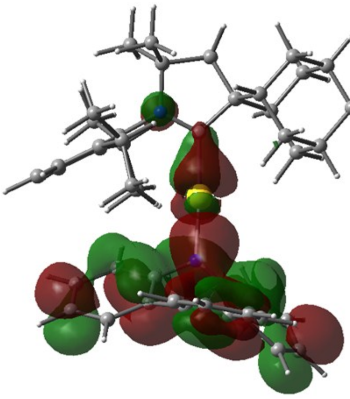 Au: 6.0%	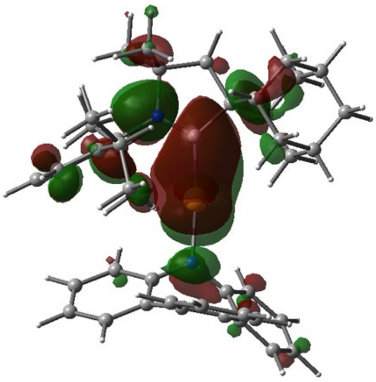 Au: 14.7%
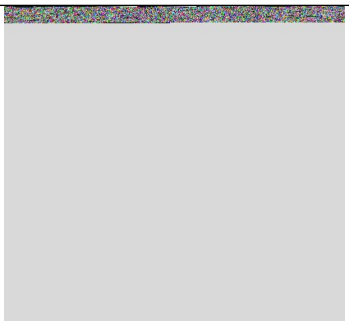 **Cu3**	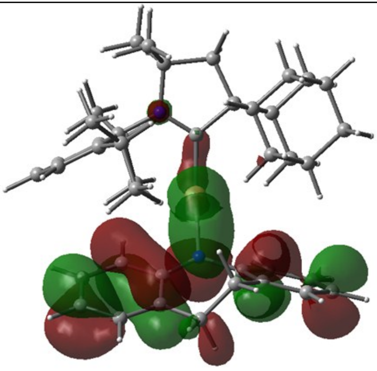 Cu: 5.1%	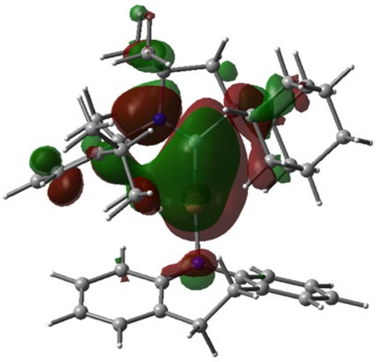 Cu: 9.1%
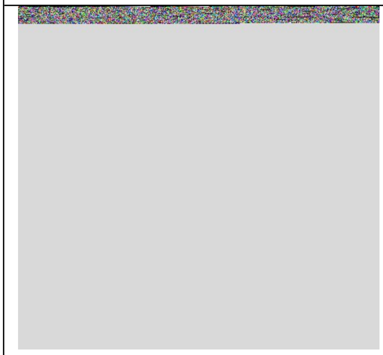 **Au3**	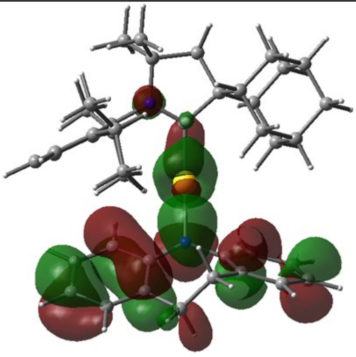 Au: 4.8%	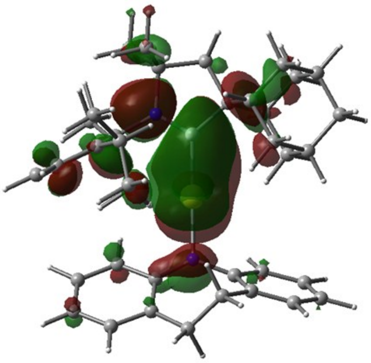 Au: 15.9%
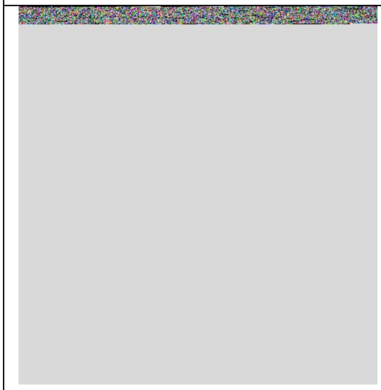 **Au4**	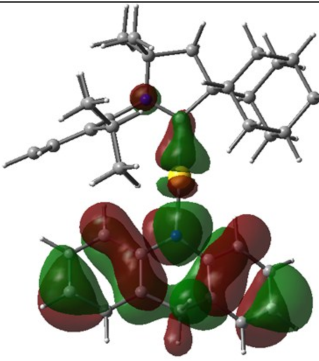 Au: 3.9%	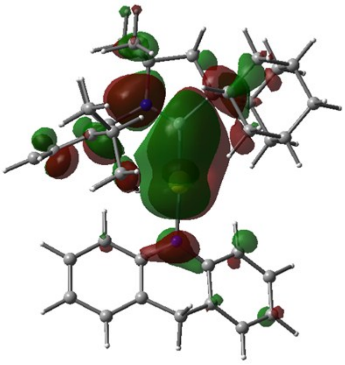 Au: 13.6%

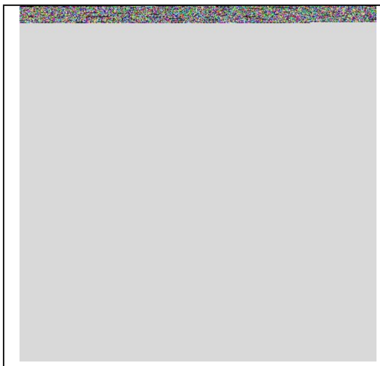 **Au5**	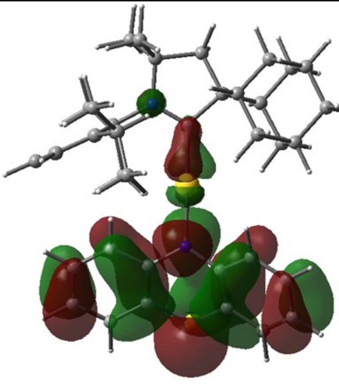 Au: 3.5%	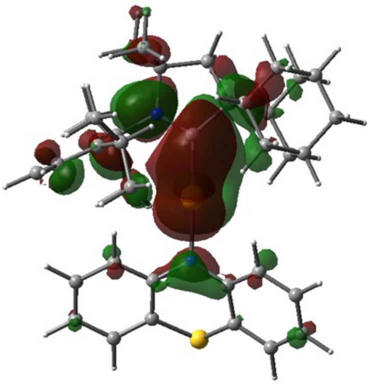 Au: 12.9%
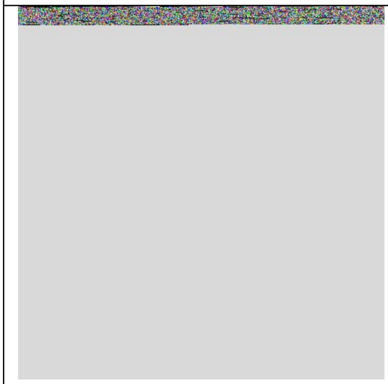 **Cu6**	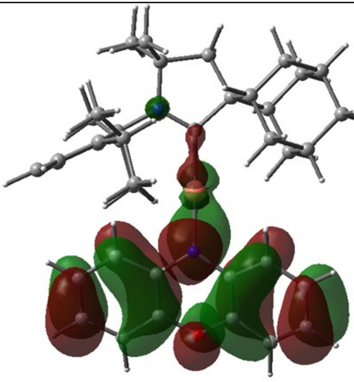 Cu: 3.1%	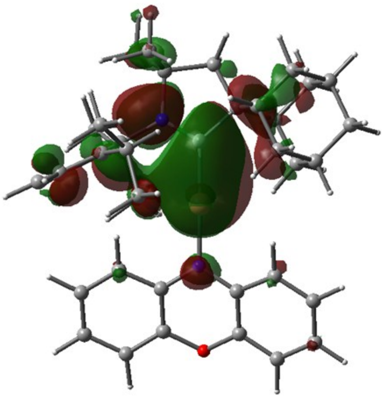 Cu: 7.8%
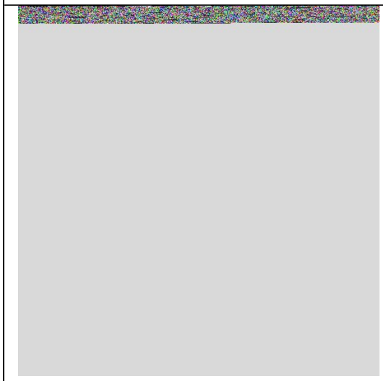 **Au6**	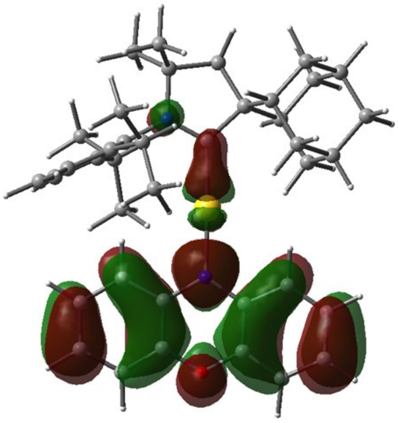 Au: 2.8%	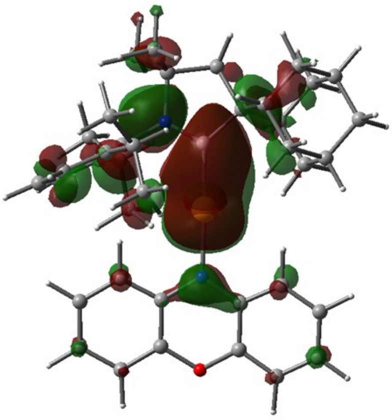 Au: 12.0%

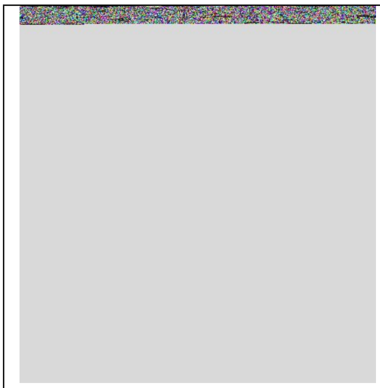 **Au8**	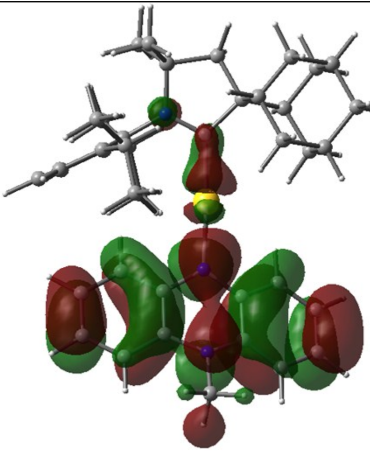 Au: 3.0%	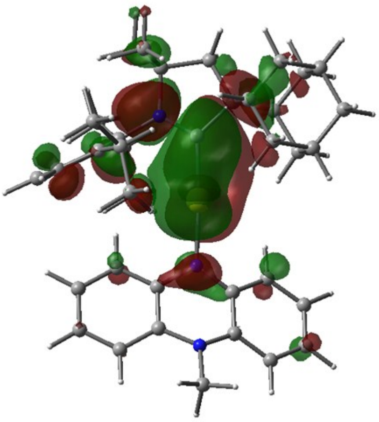 Au: 13.1%
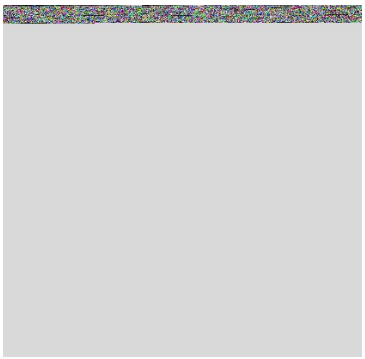 **Au7**	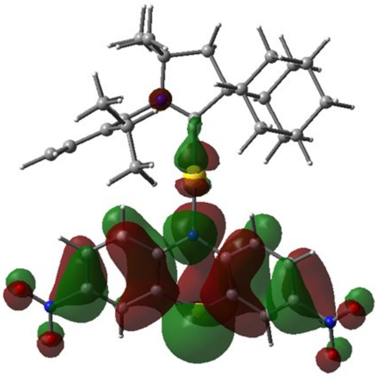 Au: 2.8%	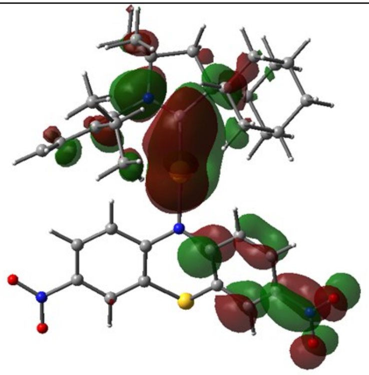 Au: 11.5% 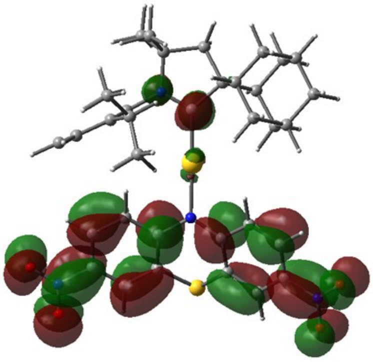 LUMO+1

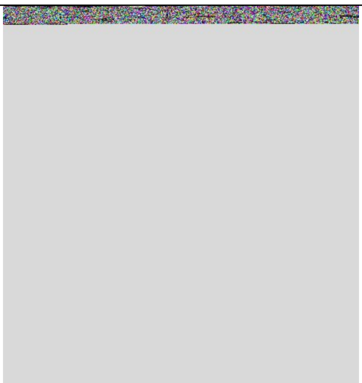 **Au8** adCAAC-Au-NPh2_NMe	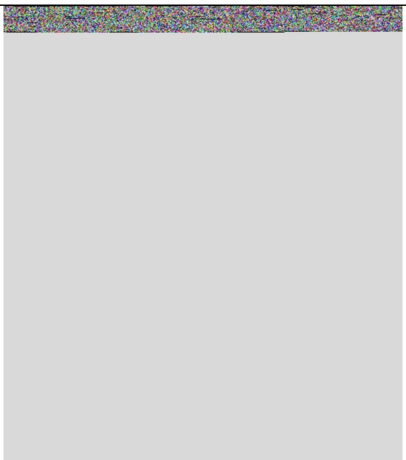 Au: 3.0%	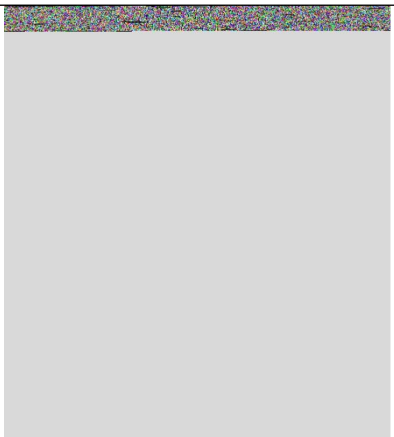 Au: 13.1%

Table S4. HOMO/LUMO gap, overlap integrals and HOMO-LUMO centroid distances

	LUMO-HOMO /eV[a]	Overlap integral of HOMO and LUMO	Centroid distance of HOMO and LUMO (Å)
**Cu1** adCAAC-Cu-NPh2_SO2	3.11	0.28	8.39
**Au1** adCAAC-Au-NPh2_SO2	3.05	0.35	8.48
**Cu2** adCAAC-Au-NPh2_Bz	3.00	0.42	7.73
**Au2** adCAAC-Cu-NPh2_Bz	2.85	0.33	7.84
**Cu3** adCAAC-Cu-NPh2_2CH2	2.69	0.31	7.77
**Au3** adCAAC-Au-NPh2_2CH2	2.76	0.41	7.71
**Au4** adCAAC-Au-NPh2_CH2	2.53	0.38	8.29
**Au5** adCAAC-Au-NPh2_S	2.45	0.37	8.74
**Cu6** adCAAC-Cu-NPh2_O	2.16	0.32	8.56
**Au6** adCAAC-Au-NPh2_O	2.22	0.40	8.43
**Au7** adCAAC-Au-NPh2_S_36NO2	2.81	0.37	7.30
**Au8** adCAAC-Au-NPh2_NMe	2.04	0.38	8.79

[a] Referenced to experimental value of CMA1[17]

Table S5. S_1 / T_1 energies, vertical excitation character and $S_0 \rightarrow S_1$ oscillator strength

Compound	S1/T1/ (ΔE_{ST} eV)	S0-S1 character	S0-S1 oscillator strength	S0-S1 charge transfer ($-e^{-1} - e^{-1}$)
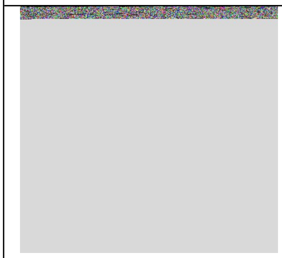 **Cu1**	3.23/3.01 (0.22)	HOMO-LUMO (96%)	0.0875	

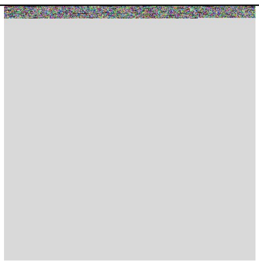 **Au1**	3.22/2.96 (0.26)	HOMO-LUMO (97%)	0.1495	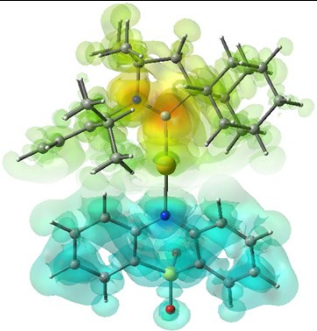
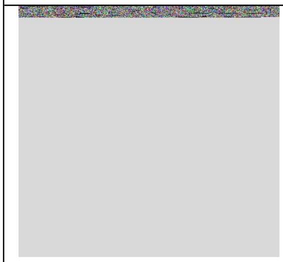 **Cu2**	2.92/2.61 (0.31)	HOMO-LUMO (94%)	0.1114	
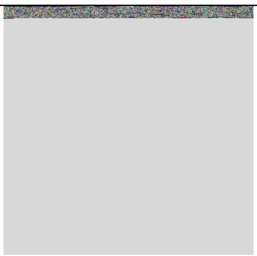 **Au2**	3.15/2.71 (0.44)	HOMO-LUMO (94%)	0.2282	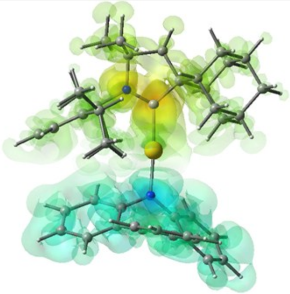
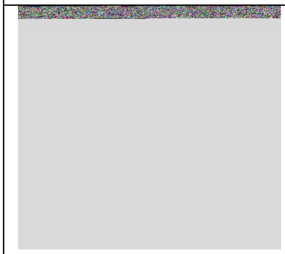 **Cu3**	2.78/2.52 (0.26)	HOMO-LUMO (96%)	0.0765	
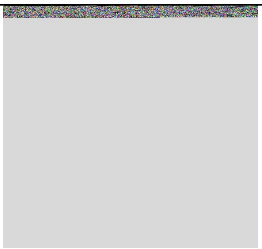 **Au3**	2.92/2.54 (0.38)	HOMO-LUMO (97%)	0.1653	

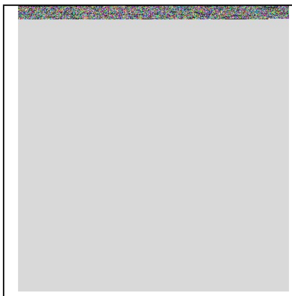 **Au4**	2.74/2.41 (0.33)	HOMO-LUMO (97%)	0.1706	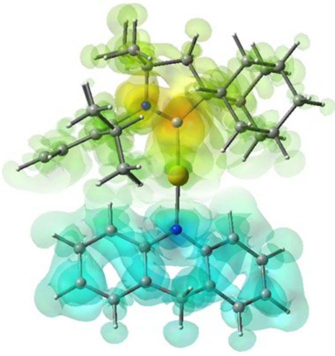
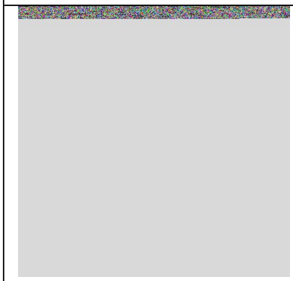 **Au5**	2.70/2.39 (0.31)	HOMO-LUMO (97%)	0.1658	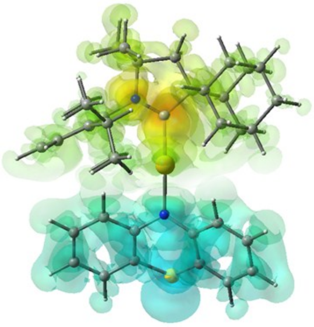
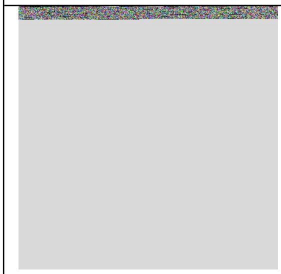 **Cu6**	2.37/2.12 (0.25)	HOMO-LUMO (98%)	0.1093	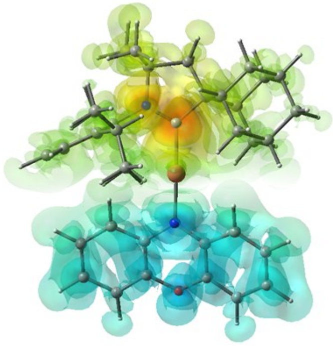
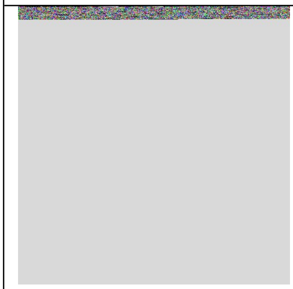 **Au6**	2.49/2.13 (0.36)	HOMO-LUMO (97%)	0.1832	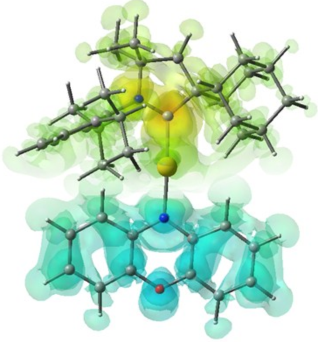

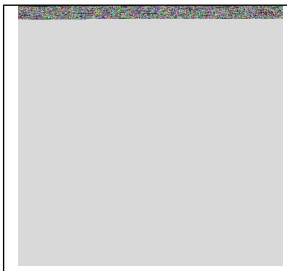 **Au7**	2.86/2.31 (0.55)	HOMO – LUMO+1 (80%)	0.2634	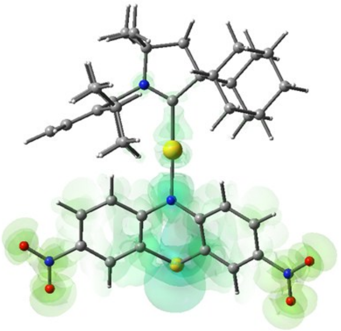
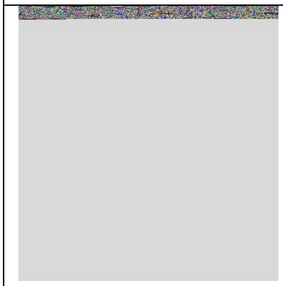 **Au8**	2.33/2.00 (0.33)	HOMO-LUMO (97%)	0.1722	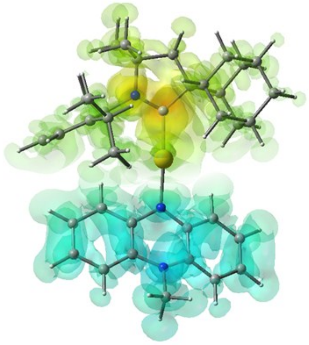

Table S6. Dipole moments in S0 and S1 states in S0 geometry

	S0	S1@S0
**Cu1**	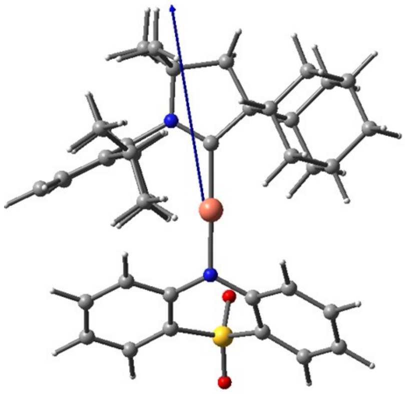 13.1D	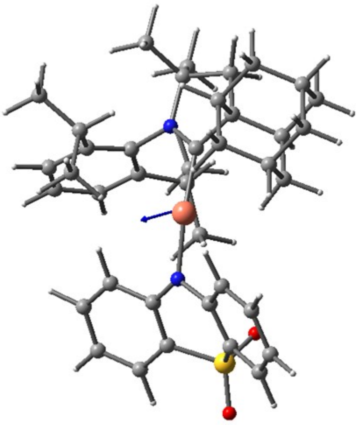 3.7D
**Au1**	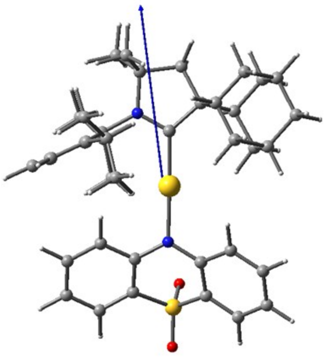 14.1D	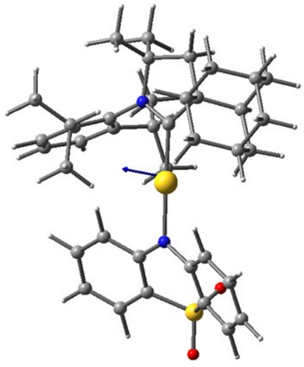 3.6D
**Cu2**	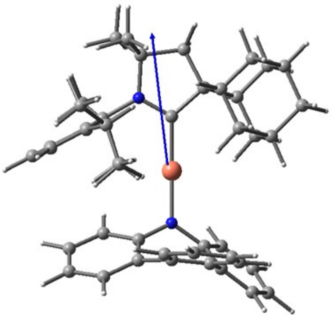 8.9D	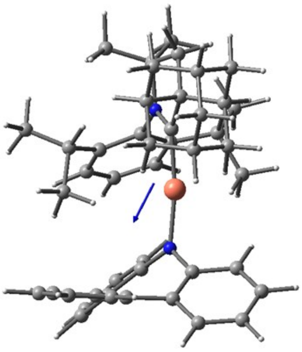 2.8D

**Au2**	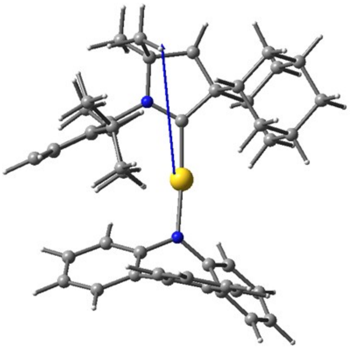 8.5D	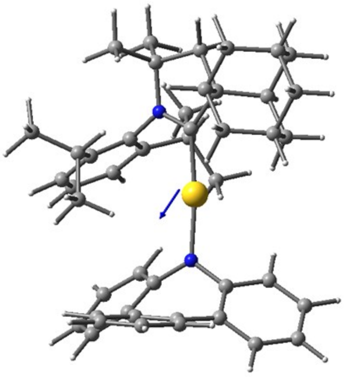 1.9D
**Cu3**	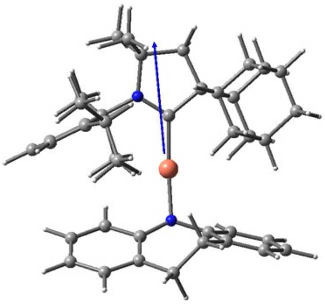 8.3D	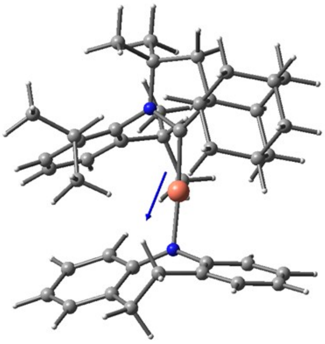 3.6D
**Au3**	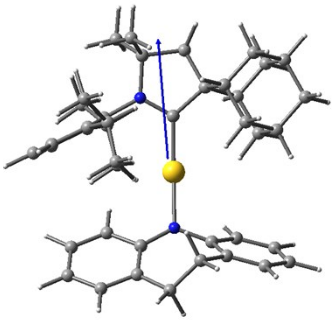 8.1D	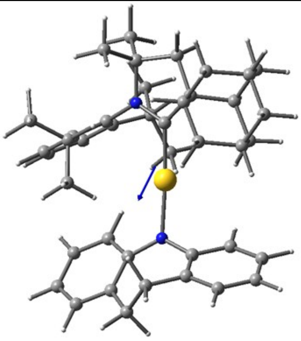 2.7D
**Au4**	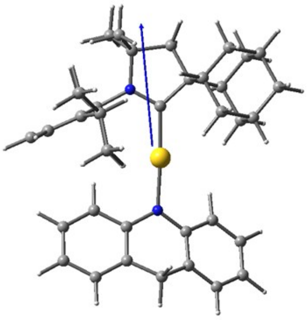 9.1D	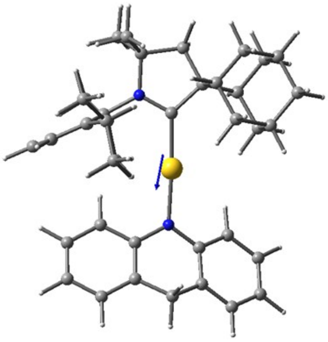 2.6D

**Au5**	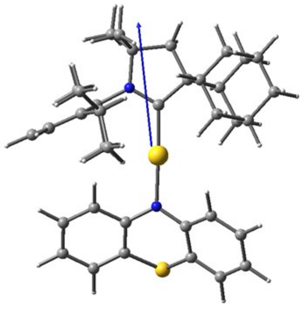 10.1D	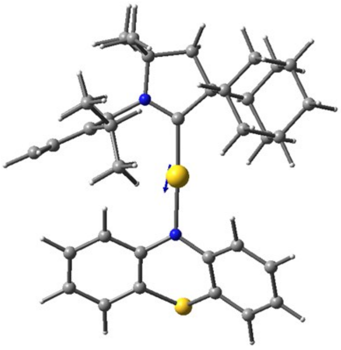 1.9D
**Cu6**	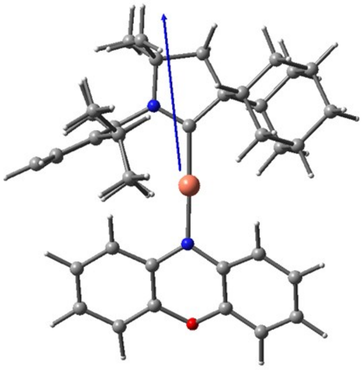 10.3D	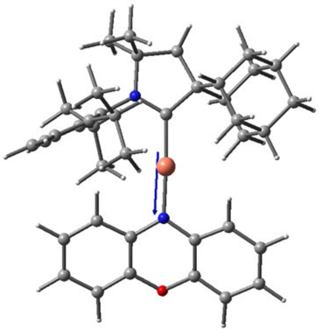 2.3D
**Au6**	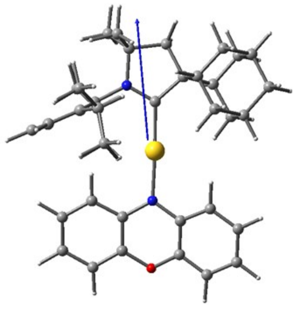 10.0D	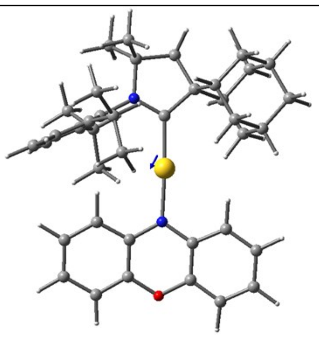 0.9D
**Au7**	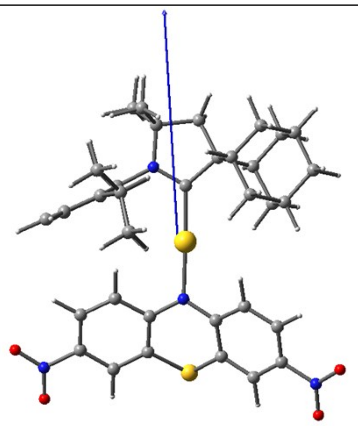 17.2D	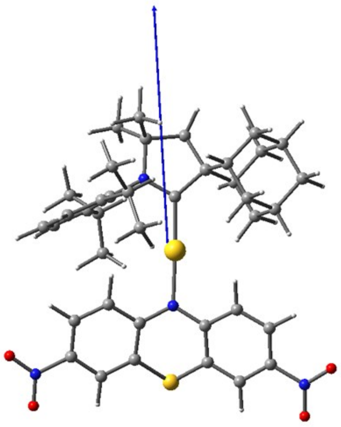 18.8D

**Au8**	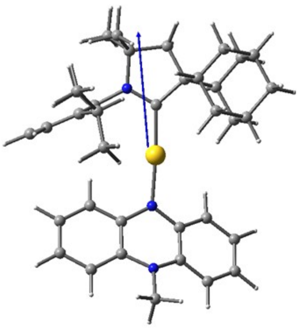 8.1D	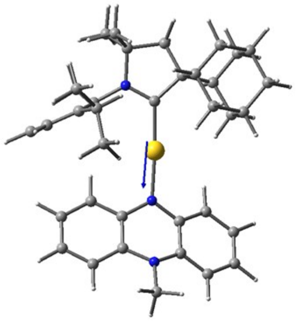 3.9D

Vacuum Processed Device Fabrication

OLED devices were fabricated by high-vacuum (10^{-7} mbar) thermal evaporation on ITO-coated glass substrates with sheet resistance of 15 Ω/□. The substrates were cleaned by sonication subsequently in a dilute solution of a non-ionic detergent, deionised water, acetone, and propan-2-ol followed by O2 plasma treatment for 10 minutes. A 1 nm layer of MoO_3 has been deposited on ITO surface as p-type dopant. A 20 nm-thick layer of 4,4',4''-Tris[(3-methylphenyl)phenylamino]triphenylamine (m-MTDATA) was used as the electron-blocking and hole-transporting layer. Dopant complex **Au3** was co-evaporated in a 1,3-bis(*N*-carbazolyl)benzene (mCP) host at a dopant concentrations of 20 wt.%. The total emissive layer thickness was kept constant at 20 nm. A 40 nm-thick layer of TPBi was used as the electron-transport layer. The cathode is formed of a 1 nm-thick layer of LiF and a 100 nm-thick layer of Al evaporated through a shadow mask to define a 4.5 mm^2 pixel area.

Solution Processed Device Fabrication

Devices were carried out by solution processing with a conventional configuration as follows: ITO/PEDOT:PSS/TFB/EML/TPBi/LiF/Al. Indium tin-oxide (ITO; WF ~ 4.8 eV) coated glass substrates were used and subsequently cleaned by sonication in acetone and 2-propanol for 10 minutes, followed by O2 plasma treatment for 10 minutes. PEDOT: PSS (Clevios CH4083, LumTech Taiwan) was spin-casted on top of the ITO under ambient conditions and annealed on a hot plate at 160°C for 20 minutes, forming a 40 nm-thick film. The PEDOT: PSS-coated substrates were then transferred to a nitrogen-filled glovebox to conduct the following solution processes. An extra hole injection layer, poly(9,9-dioctylfluorene-alt-N-(4-sec-butylphenyl)-diphenylamine) (TFB, Sigma-Aldrich) was spin-casted onto the PEDOT: PSS layer at the concentration of 15 mg/ml in toluene (Sigma-Aldrich) followed with an annealing process for 30 min at 150°C. The annealed films were spin-rinsed with

chlorobenzene (Sigma-Aldrich) to remove the residual soluble material and baked at 90°C for 10 min to remove the remaining rinsing solvent and form a compact interlayer with the thickness of around 10 nm. A 50nm-thick emitting layer of 20% Au1: PVK (or CBP) was spin-coated on top of the PEDOT:PSS from 10 mg/ml solutions in chlorobenzene, followed by post-annealing at 90 °C for 10 min to evaporate the remaining solvent. Samples were then moved into a vacuum evaporator for thermal evaporation of a 70 nm-thick TPBi layer as the ETL. Afterwards, 0.8 nm-thick LiF and 100 nm Al were deposited as the cathode. The pixel size is 4.5 mm^2.

OLED Device Characterisation

The EL spectra of the solution processed-devices were recorded using a Labsphere CDS-610 spectrometer. EL intensity was measured using a calibrated silicon photodiode with an active area of 100 mm^2, positioned vertically over the centre of the pixel, with a fixed distance between the photodiode and the pixel. A Keithley 2400 source meter measured the current-voltage characteristics of the device, while a Keithley 2000 connected to the photodiode used as a digital multiplier. OLED performance metrics were extrapolated from these on-axis measurements assuming a Lambertian emission profile.

References

[1] (a) Lavallo, V.; Canac, Y.; Prasang, C.; Donnadieu, B.; Bertrand, G. *Angew. Chem., Int. Ed.* **2005**, *44*, 5705. (b) Jazzar, R.; Dewhurst, R. D.; Bourg, J.-B.; Donnadieu, B.; Canac, Y.; Bertrand, G. *Angew. Chem., Int. Ed.* **2007**, *46*, 2899. (c) Jazzar, R.; Bourg, J.-B.; Dewhurst, R. D.; Donnadieu, B.; Bertrand, G. *J. Org. Chem.* **2007**, *72*, 3492.

[2] A. S. Romanov, M. Bochmann, *Organometallics* **2015**, *34*, 2439

[3] G. Gritzner, J. Kůta, *Electrochim. Acta* **1984**, *29*, 869.

[4] Spek, A. L., *Acta Cryst.* **2009**, *D65*, 148.

[5] Sluis, P. van der, Spek, A. L., *Acta Cryst.* **1990**, *A46*, 194.

[6] *Programs CrysAlisPro*, Oxford Diffraction Ltd., Abingdon, UK (2010).

[7] a. Sheldrick, G.M. SHELX-97 and SHELXL – Programs for crystal structure determination (SHELXS) and refinement (SHELXL), *Acta Cryst.* **2008**, *A64*, 112.

b. G.M. Sheldrick (2015) "Crystal structure refinement with SHELXL", Acta Cryst., C71, 3-8 (Open Access).

[8] F. Furche, D. Rappoport, Density functional methods for excited states: equilibrium structure and electronic spectra. In Computational Photochemistry; M. Olivuccim, Ed.;

Elsevier: Amsterdam, 2005; pp. 93–128.

[9] M. J. G. Peach, D. J. Tozer, *J. Phys. Chem. A* **2012**, *116*, 9783–9789.

[10] H.S. Yu, X. He, S.L. Li, D.G. Truhlar, *Chem. Sci*. **2016**, *7*, 5032–5051.

[11] A.S. Romanov, S.T.E. Jones, L. Yang, P.J. Conaghan, D. Di, M. Linnolahti, D. Credgington, M. Bochmann, *Adv. Optical Mater.* **2018**, 1801347.

[12] A.S. Romanov, L. Yang, S.T.E. Jones, D. Di, O.J. Morley, B.H. Drummond, A.P.M. Reponen, M. Linnolahti, D. Credgington, M. Bochmann, *Chem. Mater.* **2019**, *31*, 3613–3623.

[13] F. Weigend, M. Häser, H. Patzelt, R. Ahlrichs, *Chem. Phys. Lett.* **1998**, *294*, 143–152.

[14] F. Weigend, R. Ahlrichs, *Phys. Chem. Chem. Phys.* **2005**, *7*, 3297–3305.

[15] D. Andrae, U. Haeussermann, M. Dolg, H. Stoll, H. Preuss, *Theor. Chim. Acta* **1990**, *77*, 123–141.

[16] Gaussian 16, Revision A.03, M.J. Frisch, G.W. Trucks, H.B. Schlegel, G.E. Scuseria, M.A. Robb, J.R. Cheeseman, G. Scalmani, V. Barone, G.A. Petersson, H. Nakatsuji, X. Li, M. Caricato, A.V. Marenich, J. Bloino, B.G. Janesko, R. Gomperts, B. Mennucci, H.P. Hratchian, J.V. Ortiz, A.F. Izmaylov, J.L. Sonnenberg, D. Williams-Young, F. Ding, F. Lipparini, F. Egidi, J. Goings, B. Peng, A. Petrone, T. Henderson, D. Ranasinghe, V.G. Zakrzewski, J. Gao, N. Rega, G. Zheng, W. Liang, M. Hada, M. Ehara, K. Toyota, R. Fukuda, J. Hasegawa, M. Ishida, T. Nakajima, Y. Honda, O. Kitao, H. Nakai, T. Vreven, K. Throssell, J.A. Montgomery, Jr., J.E. Peralta, F. Ogliaro, M.J. Bearpark, J.J. Heyd, E.N. Brothers, K.N. Kudin, V.N. Staroverov, T.A. Keith, R. Kobayashi, J. Normand, K. Raghavachari, A P. Rendell, J.C. Burant, S.S. Iyengar, J. Tomasi, M. Cossi, J.M. Millam, M. Klene, C. Adamo, R. Cammi, J.W. Ochterski, R.L. Martin, K. Morokuma, O. Farkas, J.B. Foresman, D.J. Fox, Gaussian, Inc., Wallingford CT, **2016**.

[17] Di, D. ; Romanov, A. S.; Yang, L.; Richter, J. M.; Rivett, J. P. H.; Jones, S.; Thomas, T. H.; Jalebi, M. A.; Friend, R. H.; Linnolahti, M.; Bochmann, M.; Credgington, D., High-performance light emitting diodes based on carbene-metal-amides. *Science* **2017**, *356* (6334), 159-163.
